# Gene expression trees in lymphoid development

**DOI:** 10.1186/1471-2172-8-25

**Published:** 2007-10-09

**Authors:** Ivan G Costa, Stefan Roepcke, Alexander Schliep

**Affiliations:** 1 Department of Computational Molecular Biology, Max Planck Institute for Molecular Genetics, Berlin, Germany

## Abstract

**Background:**

The regulatory processes that govern cell proliferation and differentiation are central to developmental biology. Particularly well studied in this respect is the lymphoid system due to its importance for basic biology and for clinical applications. Gene expression measured in lymphoid cells in several distinguishable developmental stages helps in the elucidation of underlying molecular processes, which change gradually over time and lock cells in either the B cell, T cell or Natural Killer cell lineages. Large-scale analysis of these *gene expression trees *requires computational support for tasks ranging from visualization, querying, and finding clusters of similar genes, to answering detailed questions about the functional roles of individual genes.

**Results:**

We present the first statistical framework designed to analyze gene expression data as it is collected in the course of lymphoid development through clusters of co-expressed genes and additional heterogeneous data. We introduce dependence trees for continuous variates, which model the inherent dependencies during the differentiation process naturally as gene expression trees. Several trees are combined in a mixture model to allow inference of potentially overlapping clusters of co-expressed genes. Additionally, we predict microRNA targets.

**Conclusion:**

Computational results for several data sets from the lymphoid system demonstrate the relevance of our framework. We recover well-known biological facts and identify promising novel regulatory elements of genes and their functional assignments. The implementation of our method (licensed under the GPL) is available at .

## Background

The study of gene regulatory mechanisms controlling cell proliferation and differentiation is central in developmental biology. Because all hematopoietic cells are easily obtained as individual cells, and due to high clinical interest, the development of lymphocytes is particularly well-studied [1,2]. In mammals, all blood cells develop from pluri-potent, self-renewing hematopoietic stem cells (pHSC) of the bone marrow. In the classical model, these pHSC differentiate into common myelo-erythroid progenitors and common lymphoid progenitors [3]. The latter give rise to all cells of the adaptive immune system, including T, B and natural killer cells, which are the focus of our work.

Lymphocytes are well characterized; they can be purified by fluorescence activated cell sorting (FACS) exploiting the large variety of cell surface antigens, which appear in specific order during differentiation as the result of a linear sequence of genomic rearrangements at the T and B cell receptor loci [4,5]. Based on this, lineage-specific expression and roles of transcription factors have been studied extensively [1,2,6]. It has been shown, for example, that Gata3 is required for CD4 T cell maturation and that Runx3 silences the CD4 gene in CD8 T cells. Very recently, a new class of regulatory RNAs, microRNAs, have been identified as being involved in lymphocyte cell development [7-9].

**Figure 1:**
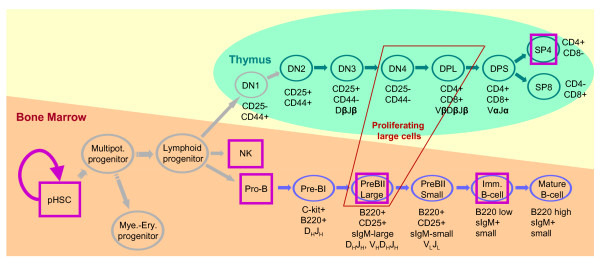
**Schematic view of lymphocyte cell development**. Developmental stages are depicted as nodes and arrows indicate transition from one stage to another, i.e. specialization. Self-renewing hematopoietic stem cells give rise to T cells in the thymus (green), B cells in the bone marrow (blue) and natural killer cells (NK) via intermediate stages. DN stands for CD4-/CD8-double negative cells, DPL for CD4+/CD8+ double positive large cells, and DPS for CD4+/CD8+ double positive small cells. Cell surface antigens and rearrangement events are partially annotated. The expression data sets investigated in this paper are marked as follows: green ovals for TCell, blue ovals for BCell, and pink boxes for LymphoidTree. We do not investigate developmental stages and transitions depicted in grey.

Several groups [4,5,10-12] have combined FACS mediated cell sorting and mRNA expression profiling to derive a more comprehensive picture of the lymphocytes in distinguishable developmental stages. Our interest focuses on these patterns of gene expression in the distinct stages of the developmental tree, the *developmental profiles *of genes; see Fig. 1 for a developmental tree. Observing such patterns, the first natural question to ask is whether further genes exhibit the same developmental profile; for example, are there other genes co-expressed with Gata3. It is reasonable to assume that genes with a prescribed pattern of expression, such as "up-regulated in proliferating cells", might be relevant for specific functions of cells in a particular stage of differentiation. Clearly, not all relevant developmental profiles are known beforehand, so clustering is the next logical step. Clustering allows us to divide genes into groups of similar developmental profiles, some of which will be irrelevant–genes expressed in all stages–others will differ in distinct branches of the developmental tree and thus indicate relevance for differentiation. Once the gamut of developmental profiles is determined, further questions can be addressed with statistical methods: which regulatory effects might cause differentiation, which subgroups of developmental stages share regulatory patterns or at which developmental stage is the difference in expression between two groups the largest. Prior work in this context relies on classical clustering methods, such as self-organizing maps [4,5], hierarchical clustering [12], or on performing tests of differential expression between cell types of interest [11]. Further studies concentrated on small-scale data, where selected genes are used to infer regulatory networks. One such study applied a state-space model to infer networks of T cell activation [13]. Troncale and colleagues adopted Petri Nets to model and infer regulatory networks of early pHSC development [14], while Basso and colleagues proposed a novel algorithm for a similar task [15].

Classical clustering relies on distance functions between developmental profiles such as correlation or Euclidean distance, which neglect the dependence structure of the developmental tree (Fig. 1). As a matter of fact, the clustering result does not change if one permutes all the variables. Biology suggests however, that the very sequence of changes does matter as this exact sequence of events is what takes a cell from pluri-potent to, say, mature B-cell. Thus we propose dependence tree models–see [16] for the discrete variate version–to model expression during the course of development. Our model assumes that the dependence of gene expression between subsequent stages is the most relevant one for identification of co-expressed genes. We assume that gene expression has been measured for a sufficient number of stages, in particular those relevant for differentiation processes, and that the cell population in a particular stage is sufficiently pure. The disagreement between reality and our assumptions is subsumed as noise, which our method can successfully deal with on simulated data. If we consider all pairwise dependencies between developmental stages our model would be equivalent to a multivariate Gaussian distribution with full covariance matrix. Due to the complexity the estimation of such models is prone to over-fitting [17,18]. The dependence tree model represents a tradeoff between methods assuming independence between variables, such as *k*-means and hierarchical clustering, and complex models, such as multivariate Gaussians, which makes estimation more robust.

With one such tree we can find genes with a specified developmental profile, for example similar to the developmental profile of *Gata*3, by ranking genes in order of decreasing likelihood under the tree. To cluster developmental profiles we combine several trees with the same topology but with distinct parameters in a classical mixture model [17]; tree topologies are taken from the biological literature. Thus we obtain a robust and flexible statistical model for clustering genome-wide mRNA expression data sets, which takes the inherent dependencies between developmental stages explicitly into account. The resulting clusters of genes sharing similar developmental expression profiles are well-suited for a subsequent search for common regulators such as transcription factors or microRNAs.

Our choice of model class is motivated by the successful application of mixtures of complex statistical models to the analysis of mRNA expression time-courses. There, models that take temporal dependencies into account, such as Splines [19,20], Autoregressive models [21] or Hidden Markov models [22], outperform simpler models, which assume independence of the variables, for example *k*-means, self-organizing maps or hierarchical clustering.

For discrete variates, dependence trees were first proposed by Chow and Liu [16], who showed that efficient computation is possible. Mixtures of trees were first proposed and applied in image recognition problems [23], where more efficient versions of the structure learning algorithm for sparse data sets became necessary. In bioinformatics, mixtures of trees were applied to infer mutation events in HIV strains [24]. We present an extension of the dependence trees to continuous variates, requiring modifications to the densities and provide a framework for robust clustering based on mixtures. To the best of our knowledge, there is no prior work on genome-scale mRNA expression analysis in which the developmental tree structure is taken into account. Both the biological application and our approach of combining tree models with mixture estimation for this purpose is novel. However, the main methodological ingredients are well-established. Our advanced statistical framework allows us to identify clusters of genes with similar developmental profiles. We detect interesting groups of genes not found using standard techniques, such as self-organizing maps [25], in developing lymphoid cells. Results on simulated data show the conditions under which our method has a technical advantage. From our clustering results we can identify plausible regulatory roles of microRNAs known to be involved in hematopoiesis. We provide a graphical user interface and a web database of clustering results; see [26] for implementations, a tutorial on how to use the tools, and a web database with the results presented below. Our findings suggest that our framework is well-suited for analysis of genome-wide expression data from detailed cell development studies.

## Results/Discussion

In the next two sections, we describe the dependence trees and how they are combined in a mixture to find groups of developmental profiles. Subsequently, we present the results of the application of our method to three lymphoid cell datasets. In the last subsection, we analyze the groups of genes, given by our mixture of dependence trees (MixDTrees) results, for common microRNA binding sites patterns, in order to gain insights into regulatory function of microRNAs.

### Dependence trees

**Figure 2:**
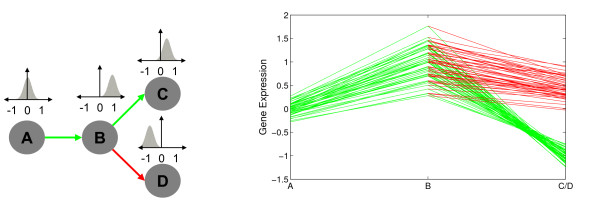
**Example of a simple developmental tree and a cluster of developmental profiles**. On the left, we depict a simple development tree, where arrows represent dependencies between variables. Above each tree variable, we depict a distribution related to it. On the right, we display the gene expression values (*y*-axis) in the distinct development stages (*x*-axis). Each line corresponds to the developmental profile of a given gene of a particular path of the tree in the left, as in a time-course plot. Distinct paths have distinct colors, in correspondence with the tree on the left. In this particular example, we have the path A, B and C in green and B and D in red. By superimposing the lines corresponding to paths B to C and B to D, we can contrast the differences in expression values of genes in these two alternative differentiation pathways.

The main assumption behind the dependence trees (DTree) is that expression levels of a particular developmental stage depend primarily on expression levels of the immediately preceding stage. For example, cf. Fig. 2, we can approximate the joint probability density function (pdf) of four random variables (*X*_*A*_, *X*_*B*_, *X*_*C*_, *X*_*D*_) by

*p *[*X*_*A*_, *X*_*B*_, *X*_*C*_, *X*_*D*_] ≈ *p *[*X*_*A*_]*p *[*X*_*B*_|*X*_*A*_]*p *[*X*_*C*_|*X*_*B*_]*p *[*X*_*D*_|*X*_*B*_].

In other words, we condition the probability of a given variable on its immediate predecessor, in accordance with the tree structure shown in Fig. 2. There, also a cluster of hypothetical genes with similar developmental profiles is depicted (Fig. 2, right). The genes display average expression in stage A, up-regulation in stage B, down-regulation in stage C and up-regulation in stage D. Furthermore, the genes have clearly distinct expression intensities, but similar relative expression changes. Genes strongly over-expressed in B are also strongly under-expressed in C and strongly expressed in D. These dependencies are reflected in the correlation between these stages. For example, A and B (or B and D) are positively correlated, and stages B and C are negatively correlated. A statistical model for such developmental profiles has to include these dependencies between subsequent stages, as it is provided by dependence trees. Let *X *= (*X*_1_, ..., *X*_*u*_, ..., *X*_*L*_) be a *L*-dimensional continuous random vector where the variable *X*_*u *_denotes the expression values of the developmental stage *u *and *x *= (*x*_1_, ..., *x*_*L*_) denotes a realization of *X *representing a developmental profile of a gene. We represent a tree by its predecessor or parent map, pa {1, ..., *L*} ↦ {1, ..., *L*} for which we assume without loss of generality that 1 *< pa*(*u*) *< u *and pa(1) = 1. Then we can write for the probability density function (pdf) of a conditional

$$p[x|\theta ]=p[x_{1}|\tau_{1}]\prod_{u=2}^{L}p[x_{u}|x_{\text{pa}(u)},\tau_{u}].$$

We denote the model parameters by *θ *= (*τ*_1_, ..., *τ*_*u*_, ... *τ*_*L*_) and the DTree by the tuple (*X*, pa, *θ*). Note, that a DTree can also be viewed as an approximation of the joint distribution of a *L*-dimensional continuous random vector by a product of *L *- 1 second order distributions [16].

We use conditional Gaussian density functions [27] as conditional densities, denoted by *p *[*x*_*u*_|*x*_pa(*u*)_, *τ*_*u*_] in Eq. 2. Hence, for a given developmental profile *x *and a non-root developmental stage *u *with pa(*u*) = *v*, the pdf takes the form

$$p[x_{u}|x_{v},\tau_{u}]={\left(\sqrt{2\pi }\sigma_{u|v}\right)}^{-1}\exp\left(\frac{-{\left(x_{u}-\mu_{u}-w_{u|v}(x_{v}-\mu_{v})\right)}^{2}}{2\sigma_{u|v}^{2}}\right),$$

where *τ*_*u *_= (*μ*_*u*_, *w*_*u*|*v*_, $\sigma_{u|v}^{2}$) are the parameters for one conditional density in the model.

For a given expression data set consisting of measurements for *N *genes at *L *developmental stages, let *x*_*i *_= (*x*_*i*1_, ..., *x*_*iu*_, ..., *x*_*iL*_) be the developmental profile of gene *i*, and *x*_*iu *_be the expression value of the gene *i *in development stage *u *for 1 ≤ *i *≤ *N *and 1 ≤ *u *≤ *L*. As derived in the Protocol in the Additional data file 1, the maximum likelihood estimates (MLE) for the parameters of the conditional Gaussian are

$${\hat{\mu }}_{u}=(\sum_{i=1}^{N}x_{iu})/N,$$

$${\hat{w}}_{u|v}=\frac{{\hat{\sigma }}_{uv}}{{\hat{\sigma }}_{v}^{2}},\text{ and}$$

$${\hat{\sigma }}_{u|v}^{2}={\hat{\sigma }}_{u}^{2}-{\hat{w}}_{u|v}^{2}{\hat{\sigma }}_{v}^{2}.$$

These terms can be computed from the sufficient statistics

$${\hat{\sigma }}_{u}^{2}=(\sum_{i=1}^{N}{\left(x_{iu}-{\hat{\mu }}_{u}\right)}^{2})/N,\text{ and}$$

$${\hat{\sigma }}_{uv}=(\sum_{i=1}^{N}(x_{iu}-{\hat{\mu }}_{u})(x_{iv}-{\hat{\mu }}_{v}))/N.$$

The conditional normal distribution can be seen as estimating a linear fit between *X*_*u *_and *X*_*v*_, where *w*_*u*|*v*_*> *0 indicates a positive linear correlation and *w*_*u*|*v*_*<*0 a negative linear correlation between variables; *w*_*u*|*v *_= 0 if the variables are independent. Furthermore, *w*_*u*|*v *_and $\sigma_{u|v}^{2}$ are related because the better the linear fit the smaller the variance. For the special case of the root (recall pa(1) = 1), *w*_1|1 _is set to zero, and the conditional density is effectively a univariate normal. In total, the model has 3*L *- 1 free parameters.

A very simple, but useful application, is to query the developmental profiles from a data set with a tree model. By defining the model parameters in an interactive manner, we can compute the likelihood (Eq. 2) of all expression profiles *x*_*i*_. rank them accordingly, and list the *m *most likely profiles (see [26] for the tool description and tutorial). This interactive tool allows biological experts to find genes following a developmental profile of interest.

Returning to the example in Fig. 2, the model estimates given the tree and developmental profiles are

$$\begin{array}{ccccc} \tau_{A} & = & \left({\hat{\mu }}_{A},{\hat{w}}_{A},{\hat{\sigma }}_{A}^{2}\right) & = & (-0.01,0,0.02),\\ \tau_{B} & = & \left({\hat{\mu }}_{B},{\hat{w}}_{B|A},{\hat{\sigma }}_{B|A}^{2}\right) & = & (0.97,2.2,0.02),\\ \tau_{C} & = & \left({\hat{\mu }}_{C},{\hat{w}}_{C|B},{\hat{\sigma }}_{C|B}^{2}\right) & = & (-0.99,-0.3,0.01),\text{ and}\\ \tau_{D} & = & \left({\hat{\mu }}_{D},{\hat{w}}_{D|B},{\hat{\sigma }}_{D|B}^{2}\right) & = & (0.45,0.53,0.01). \end{array}$$

As expected, *w*_*B*|*A *_and *w*_*D*|*B *_are positive, indicating a linear dependence between these variables. On the other hand *w*_*C*|*B *_is negative.

### Mixtures of dependence trees

**Figure 3:**
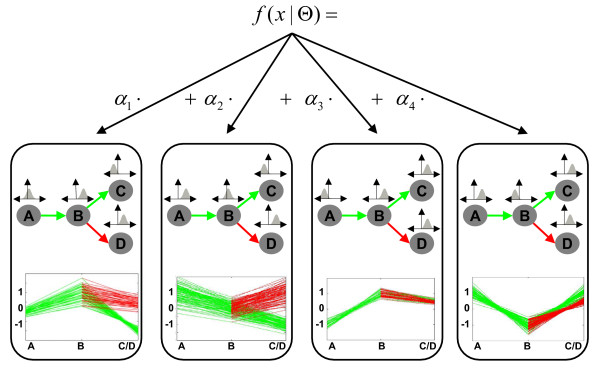
**Example of a mixture of four dependence trees with the topology defined in Fig. 2**. Each of the trees models distinct developmental profiles found in an example data set. Furthermore, clusters may have distinct sizes proportional to their *α*_*i*_'s. Note also that it is not necessary that clusters have distinct expression values in branching stages. For example, stages *C *and *D *have similar expression values for cluster 3 and 4. This can be interpreted as the genes being equally expressed in the two alternative lineages.

In order to find clusters of co-expressed genes, we combine several dependence trees (DTree) in a mixture. Each DTree is a representation of a cluster or group of genes with similar developmental profiles; that is, each DTree models distinct patterns of gene expression in the course of development (see Fig. 3 for an example). The differentiation of cells is conveniently represented as a developmental tree and the structure or topology of this tree is well-known for most data sets under investigation. Consequently, all trees in a mixture share the same topology. A mixture of dependence trees accommodates overlapping clusters while reflecting the inherent dependencies between stages. Throughout this paper we refer to the presented method as well as to the resulting model as MixDTrees.

More formally, we combine a set of *K *DTrees in a mixture model $f(x|\Theta )=\sum_{k=1}^{K}\alpha_{k}p[x|\theta_{k}]$, where Θ = (*θ*_1_, ..., *θ*_*K*_, *α*_1_, ..., *α*_*K*_), *θ*_*k *_denotes the parameters of the *k*-th DTree and *α*_*k *_is proportional to the number of developmental profiles assigned to the *k*-th Dtree; as usual *α*_*k *_≥ 0 and $\sum_{k=1}^{K}\alpha_{k}=1$. To avoid over-fitting of the tree models, in particular for components with low component priors *α*_*k*_–that is, a small number of assigned genes–we propose a maximum-a-posteriori (MAP) approach, which regularizes the estimates from Eq. 5 and Eq. 6. Given this preferable characteristic, MAP estimates are used in all MixDTrees experiments, unless otherwise stated. Note also, the parameters of the mixture are estimated with the Expectation-Maximization (EM) algorithm [28] (see Methods section for EM and MAP details).

As stated in the introduction, the problem approached here is closely related to gene expression time-course analysis. There is a vast amount of literature on models and clustering methods suitable for time-courses [18-22,29,30]. Lately, attention has been given to the fact that these time-courses have usually few time points [31,32], a characteristic previously ignored. This aspect is also essential to our application, since the number of distinguishable developmental stages is usually small, for example at most seven in our data sets. Note that a single chain of subsequent development stages, such as the stages of B-cell differentiation in Fig. 1, is by definition a tree. While dependence trees are indeed also suitable for time-courses, the complex dependency structures necessary due to branching of the developmental tree into distinct lineages prevents the use of time-course models, as there is no effective way of incorporating the necessary extensions into these models [19,22]. In the context of mixtures, our method represents an alternative to the parameterization of the covariance matrix of a mixture of multivariate Gaussians [17]. With MLE, the dependence tree model essentially imputes zeros in the covariance matrix reducing the number of parameters to the order of *L*. If we would consider all the covariances between observations for *L *developmental stages; it would be straightforward to represent the data distribution by a *L*-variate Gaussian model with full covariance matrix. However, the estimates for the *L*^2 ^parameters are often unreliable even for small values of *L *and the parameter estimation is prone to over-fit to outliers often found in noisy and scarce data. In fact, mixtures of Gaussians with full covariance matrix were outperformed by simpler parameterizations of the covariance matrices in the context of gene expression time courses [18].

### Application in lymphocyte cell development

We apply our method to obtain MixDTrees for the data sets TCell, BCell, LymphoidTree, and SIM (see Methods section for details) and compare our clustering results to previous work. Our data is complemented with information from OMIM [33], the Gene Ontology database [34] and from literature. For TCell and BCell, we use the same number of clusters as Hoffmann and colleagues (20) [4,5,35] and for LymphoidTree we apply the BIC criterion [36] (see Fig. S4 in Additional data file 2), which also resulted in an optimal choice of 20 clusters. As discussed in Dependence trees section, a simple way to check for similarities in the expression between developmental stages is to compute the correlation matrix of the data set at hand (see Mixtures of dependence trees estimation section).

### T cell development (TCell)

**Figure 4:**
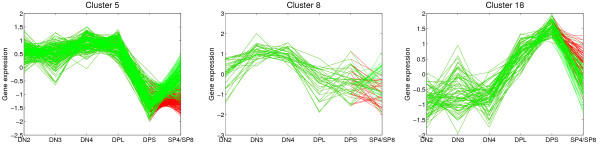
**Selected clusters from MixDTrees on Tcell**. We depict the clusters 5, 8 and 18 found in TCell, expression values on the y-axis, and cell types on the x-axis. Lines corresponding to developmental profile values between stages DN2, DN3, DN4, DPL, DPS and SP4 are in green and between DPS and SP8 in red.

TCell is a gene expression data set from seven differentiation stages of the T cell development (see Methods section and Fig. 1 for details). The only branch in this tree is the final differentiation of DPS precursors into CD4 single positive SP4 cells and CD8 single positive SP8 cells. Most clusters show a distinctive pattern of differential expression along the developmental path but do not differ between SP4 and SP8 cells (clusters 4, 7, 11, 13, 14, 15, 16, 19, and 20). The most drastic changes occur at the DPL stage in which the cells are proliferating and subsequently start to rearrange the TCR*α*-locus. This is also reflected in the overall correlation matrix (Table S1 in Additional data file 3). Although the expression values of all neighboring stages are positively correlated, the correlation between the DPL stage and the DPS stage is much smaller in comparison to the double negative stages, all of which are relatively highly correlated. The correlation matrix suggests that SP4 and SP8 cells are more similar to each other than to their precursor DPS cells, which is expected since the two types of mature T cells share many cellular functions [4]. The largest differences with respect to SP4 and SP8 are found in clusters 5 and 18 (Fig. 4). In cluster 5, cell-cycle genes are clearly enriched. In contrast, cluster 18 mainly contains regulatory proteins involved in transcription and signaling (see Fig. 4).

Hoffmann and colleagues used self-organizing maps (SOM) to cluster the expression profiles [4,5,35]. From now on, we refer to Hoffman and colleagues' results simply as SOM. In our analysis we observe clusters with similar developmental profiles, which we define as the average over the gene expression profiles of a cluster. As expected, there is not a one-to-one relationship between the two clusterings. While the single gene profiles are similar since we used analogous normalization and filtering procedures (see Methods section), the actual gene clusterings differ (see Table S12 in Additional data file 3). An objective assessment of clustering quality on developmental data is impossible due to lack of benchmarking data. Furthermore, there is no agreement in the literature on a methodology to validate clustering results [37]. In order to demonstrate that our method is able to extract additional biological information, we concentrate our discussion on clusters of distinct developmental profiles that could not be detected by SOM [4]. For such a cluster we assign functions to genes using the GO term annotation and complementary literature. Ideally, the functions of all genes of the cluster would match the cellular processes of the particular developmental stage at which these genes are over-expressed. Additionally, if some of these genes are of unknown function then the developmental profile can help to generate hypotheses about their functional role. In our analysis we find that genes of cluster 8 are over-expressed in DN3 and DN4 cells, a developmental profile that has not been previously discovered (Fig. 4). With SOM, the genes of this cluster are dispersed over the two clusters (see Table S12 in Additional data file 3). Out of the 30 genes of cluster 8 seven are related to vesicle transport, or to the Golgi/ER system. Additionally, we find five cell-cycle related genes, three involved in mitochondrial function, and seven genes of other functions, which are mainly involved in signaling. These findings agree with the functions of DN3 and DN4 cells, which is the transport of precursor receptor molecules to the cell surface membrane and the initiation of proliferation. This demonstrates that our method is able to identify functionally relevant gene sets even if the expression changes are not as large as for the DPL stage, for example. The complete results, including gene expression plots, analysis of GO-term and microRNA enrichment, can be found in our web database [26].

### B cell development (BCell)

**Figure 5:**
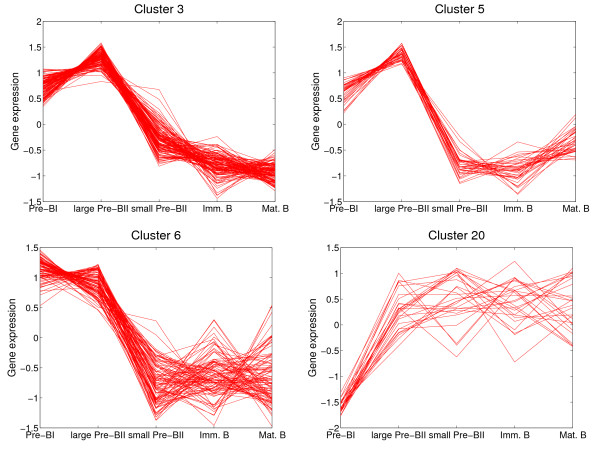
**Selected clusters from from MixDTrees on Bcell**. We depict clusters 3, 5, 6 and 20 found in BCell, expression values on the y-axis, and cell types on the x-axis. Lines corresponding to developmental profile values between between all stages are in red.

In a similar approach to the TCell study, we investigated gene expression for five consecutive stages during B cell development (see Methods section and [4,5] for details). The correlation matrix of BCell suggests dependencies between gene expression values of successive stages, with the largest correlation between pre-BI and large pre-BII cells and between immature and mature B cells (see Table S2 in Additional data file 2). When we compare, as in the TCell set, our clustering results to those of Hoffmann and colleagues [4], we observe similar average developmental profiles although the contingency table indicates differences in the cluster compositions (Table S13 in Additional data file 3). Clusters 3, 5 and 6, for example, contain genes that are up-regulated in pre-BI and large pre-BII cells and down-regulated in later developmental stages (Fig. 5). Consistent with the phenotype of these cells, the function assigned to the genes of this cluster are mainly related to proliferation. GO categories that are associated with mitosis, cell-cycle and chromatin remodeling are clearly overrepresented in these clusters (see our web database [26]).

Cluster 20 shows an average developmental profile that was not detected with SOM [4,5]. The genes of this cluster are down-regulated in pre-BI cells, in which the first rearrangement of the *D*^*H *^and *J*^*H*^segments on the *H *chain loci has taken place, and up-regulated in all the following developmental stages (Fig. 5). With SOM [4], these 23 genes are found distributed over the four clusters 11, 13, 14 and 17 (Table S12 in Additional data file 3). The most palpable common function of many cluster 20 genes is the regulation of survival and apoptosis during B cell development. The gene products *Nfkbia*, *Traf5 *and the Src-family protein tyrosine kinases *Lyn *and *Syk *are known regulators of NF-kappa B activity, which in turn has been found to be involved in B cell fate decision and survival [38-40]. Similarly, Krupel-like factor 2 (*Klf2*) protects cells against TNF-alpha induced apoptosis [41]. Furthermore, *Icam-2 *and *Rhoh*, whose encoding genes are two other members of cluster 20, regulate the adhesiveness of primary B cells depending on their activation state and protect them from apoptosis [33,42].

### Lymphoid tree (LymphoidTree)

**Figure 6:**
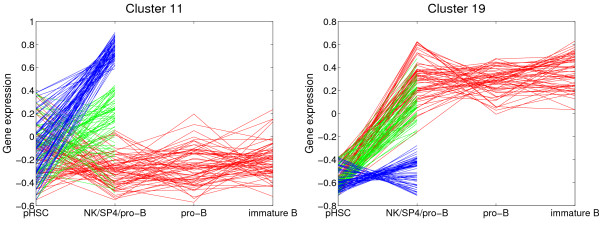
**Selected clusters from from MixDTrees on LymphoidTree**. We depict clusters 11 and 19 found in LymphoidTree, expression values on the y-axis, and cell types on the x-axis. Lines corresponding to developmental profile values between stages HSC, pro-B, pre-B and immature B cell are in read, between HSC and NK cells in blue, and between HSC and SP4 cells in green.

LymphoidTree combines data sets of several studies [10-12], and the resulting tree contains expression measurements from lymphoid cells of six developmental stages, namely hematopoietic stem cells, pro-B, pre-B, and immature B cells, mature SP4 T cells, and natural killer (NK) cells. This integration of data is possible because the studies were carried out on the same array platform. Although the developmental tree is far less detailed compared to TCell and BCell, we still gain insights on differences between the cell lineages. As expected, the correlation matrix shows that the expression patterns of the three B cell stages are more highly correlated among each other then expression patterns of different lineages. Moreover, the overall expression of SP4 cells and NK cells is positively correlated. The resulting clusters provide a basis to hypothesize about early developmental decisions and suggest target genes for further investigations. For example cluster 11 contains genes that are strongly up-regulated in NK cells, weakly induced in the SP4 cells and not expressed in the precursor B cells (Fig. 6). Many of the cluster 11 genes are well known to be expressed in NK cells, as for example the cell surface receptor genes *Cd244*, *Klra1*, and *Crtam *[33,43]. Among the lesser known genes is the one that codes for the Pu.1 related transcription factor SpiC, which has already been found to be temporarily expressed during B cell development [44]. In contrast, cluster 19 contains genes that are up-regulated in SP4 cells and in all B cell precursors but not in NK cells (Fig. 6). Important functions during B and T cell maturation are reflected by genes in this cluster, like the bruton tyrosine kinase *Btk*, the transcription factor *Pou2af1*, which is involved in immunoglobulin gene regulation, and the DNA repair genes *Trp53bp1 *and *Pnkp *[33].

### Simulated data (SIM)

We demonstrate with simulated data that our novel method outperforms established methods, such as SOM, *k*-means and mixture of Gaussians, when inferring tree components in complex mixtures for varying levels of dependence between the individual variates. The dependence is reflected in the magnitude of *w*_*u*|*v*, *k *_(Eq. 5) of a tree. By sampling these parameters from different intervals, [-*ε*, *ε *], [-0.5, 0.5], [-1, 1], [-1.0, -0.5] ∪ [0.5, 1] and [-1, -1 + *ε*] ∪ [1 – *ε*, 1], we can create mixtures with components ranging from independent models to highly dependent ones. We generate a data set for each sampled mixture. We used MixDTrees, mixture of Gaussians, *k*-means and SOM to compute clusters, which we can compare to the classes used in data generation to compute specificity and sensitivity of the clustering solutions. Method performance is evaluated with a paired *t*-test. Details are given in Methods section.

**Figure 7:**
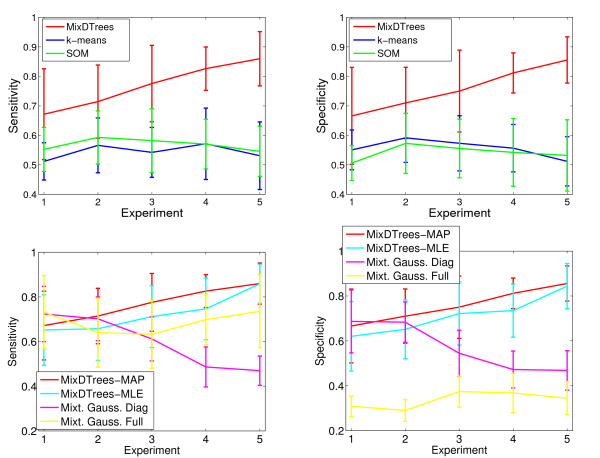
**Results of SIM**. We display the mean sensitivity (left plots) and mean specificity (right plots) against five experimental settings: (1) *w*_*u*|*v*, *k *_∈ [-*ε*, *ε *] (independent data), (2) *w*_*u*|*v*, *k *_∈ [-0.5, 0.5], (3) *w*_*u*|*v*, *k *_∈ [-1, 1], (4) *w*_*u*|*v*, *k *_∈ [-1.0, -0.5] ∪ [0.5, 1] and (5) *w*_*u*|*v*, *k *_∈ [-1, -1 + *ε*] ∪ [1 – *ε*, 1]. The dependence increases with experiment number. On the top plots, *k*-means results are displayed in blue, SOM in green and mixture of dependence trees with MAP estimation (MixDTrees) in red. On the bottom plots, mixture of Gaussians with full covariance matrices are displayed in yellow, mixture of Gaussians with diagonal covariance matrices in purple, Mixture of dependence trees with MLE estimation in light blue (MixDTrees-MLE) and mixture of dependence trees with MAP estimation (MixDTrees-MAP) in red.

We observe that the MixDTrees with MAP estimates (MixDTrees-MAP) have a higher specificity and sensitivity than *k*-means and SOM in all experimental settings (Fig. 7 top) (*p*-value below 0.005). In the (almost) independent case (*w*_*u*|*v*, *k *_∈ [-*ε*, *ε*]), this is not expected, since the data agrees well with the assumptions of *k*-means and SOM. This also explains the large standard deviations of MixDTrees-MAP in that case. As expected, the MixDTrees-MAP clearly improves the cluster recovery in settings with pronounced dependence structure, while the performance of *k*-means and SOM deteriorates slightly. In comparison to others mixture model methods (Fig. 7 bottom), MixDTrees-MAP also obtains a significantly higher specificity and sensitivity in almost all experimental settings. The mixture of Gaussians with diagonal covariance matrices performs well in the independent case (1), which meets the model assumptions, but it has poor results in experiments with higher dependence (*p*-values below 0.05 for settings 3, 4 and 5). The mixture of Gaussians with full covariance matrix (MG-Full) has a reasonable sensitivity in all settings, but very poor specificity (*p*-value below 0.05 in settings 3, 4 and 5 for specificity and in all settings for specificity). The reason for these results is that MG-Full tends to populate some clusters with few data points, a problem known as spurious local maxima [17]. Note that we use a MAP estimate of MG-Full to mitigate this problem. Even though there are other methods for detection of spurious local maxima in MG-Full, which could lead to better specificity, this would require extensions of the EM method, and consequently slower convergence [17]. On the other hand, MixDTrees, which has a lower computational running time than MG-Full, achieves good results without the need of any extension. MixDTrees with MLE estimates (MixDTrees-MLE) has good overall performance, but is outperformed by MixDTrees-MAP in all cases, except experimental settings 1 and 5 (*p*-value below 0.05 for settings 2, 3 and 4). In experimental setting 5, where data is highly dependent, by definition, both methods work similarly well. Nevertheless, such high dependency would never be found in real data sets, since noise in the data obfuscates dependencies between variables. Additionally, we performed further experiments with simulated data to evaluate the robustness of the method with respect to noise (see Additional data file 1). There, MT-MAP maintains good sensitivity and specificity of cluster recovery even for high noise levels.

This demonstrates that the MixDTrees is a superior alternative to SOM and *k*-means in all cases. In relation to other mixture models, MixDTrees represents a good tradeoff between a complex model class such as multivariate Gaussian with full covariance matrices and the simple Gaussian with diagonal covariance matrices. Furthermore, MAP estimates of the MixDTrees represent a more robust alternative to the MLE counterpart.

### MicroRNA target discovery

LympMIR contains a set of 17 microRNAs that are potentially involved in lymphocyte cell development (for details see Methods section). It has been proposed that microRNAs bind target mRNAs specifically via base pairing, which subsequently leads to interference with the translational machinery or mRNA degradation, and thus can control whole groups of genes simultaneously [45]. Recent microarray studies have demonstrated that the microRNA expression negatively correlates with mRNA target expression in a tissue specific manner [46-48].

**Figure 8:**
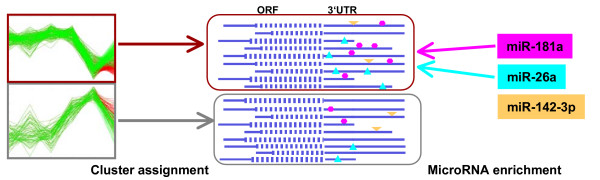
**Strategy to identify enriched microRNAs**. Strategy to identify microRNAs and their target genes overrepresented in groups of co-expressed genes (indicated left) as part of a post-transcriptional regulatory mechanism. In the middle mRNAs clustered according to our mixture results are depicted and potential microRNA binding sites in their 3'UTRs are symbolized.

Having identified a cluster of co-expressed genes during lymphoid development we ask whether a certain microRNA could be a potential regulator of this cluster (see Fig. 8). For this task we first obtain lists of potential target genes for each microRNA from the miRBase Targets database [49], which contains predictions made by sequence based methods. Given our clustering results, we use the statistic of the Chi-Square Test [50] to obtain a list of microRNAs, whose potential targets are overrepresented in a cluster. This is an analogous approach to finding Gene Ontology [51] terms over-represented in a cluster of genes. Given a set of *n *genes, we count the number *c *of genes in a given cluster, the number *t *of genes identified as targets for a given microRNA and the number *h *of genes that are both in the cluster and are targets of the microRNA. The resulting *p*-value reflects the statistical significance of observing a count *h*, given *n*, *c *and *t*. A lower *p*-value indicates a higher "microRNA enrichment", and, consequently, a better result. By choosing a *p*-value cutoff, we can construct a list of enriched microRNAs for each cluster as well as a list of target genes related to the enriched microRNAs. Note, that the statistics for microRNA-binding are not well developed; intricate dependencies introduced by sequence similarities among the microRNAs and the target genes exist and complicate the analysis. As we also consider a manually selected set of microRNAs, we choose a somewhat relaxed *p*-value cutoff, foregoing multiple testing corrections [52], followed by a careful biological evaluation. For the following discussions we restrict our result set to clusters that contain at least four target genes in total.

In summary, in TCell our target prediction scheme detects significant enrichment for eleven out of the 17 initial microRNAs in four out of the 20 clusters (Table 1). In these four clusters we detect in total 35 candidate target genes, which is a considerable reduction of the set of 229 targets that have been predicted by sequence based methods alone [49]. For BCell these numbers are respectively, eleven out of the 17 microRNAs, four out of the 20 clusters, and 29 out of the 273 predicted targets (Table 1). In particular, we find the five microRNA families miR-15, miR-181, miR-221, miR-26, and miR-142-3p to be enriched in both TCell and BCell by our criterion. See Table S6 in Additional data file 3 for microRNA enrichment in LymphoidTree and Table S7, Table S8, Table S9, for *p*-values of microRNA enrichment of all data sets. As mentioned earlier, the BCell clusters 3, 5, and 6 show a similar expression profile. We find that cluster 5 of the results of the TCell set overlaps substantially with clusters 3 and 5 of BCell (Table 1). In TCell cluster 5 we find miR-15a, miR-181a, miR-26a, miR-24, and miR-221 as potential regulators and 20 potential target genes, seven of which are also present among the 18 BCell candidate genes of clusters 3 and 5. The developmental profiles of the clusters of both lineages show strikingly analogous phenotypical features, namely up-regulation in the proliferating large cell populations (DN4, DPL, large pre-BII) and from then on strict down-regulation. In TCell cluster 5 there are eight genes and in the BCell clusters 3 and 5 there are nine target genes that are known to be involved in DNA metabolism, cell-cycle and mitosis (Table 1). This suggests a regulatory role for the identified microRNAs in reducing the transcript levels of genes that are important for cell proliferation. This is supported by the fact that a similar role for microRNA was found in Drosophila germline stem cells [53].

**Table 1:** List of LympMIR enriched in the clusters from MixDTrees on data sets TCell and BCell

Cluster ID	MicroRNA	Target Genes
TCell 3	miR-222	*Elovl6, Nme1, Rcn1, Rps3*
TCell 5	miR-15a^1^, miR-181a^2^, miR-221^3^,	*2410015N17Rik*^4^, *Alad*^1,4^, *Atpif1*^1,5^, ***Aurkb***^2^, ***Cdc25a***^1^, ***Chek1***^1^
	miR-24^4^, miR-26a^5^	***Cks1b***^2,4^, ***Cks2***^5^, *Eed*^2^, ***H2afx***^4^, *Kpnb1*^3^, ***Mcm5***^3^, *Nasp*^3,5^, *Pex7*^2^, *Psmd12*^2^, *Ranbp5*^2^, *Rars*^1^, ***Tk1***^3^, *Trip13*^1^, *Uchl5*^5^
TCell 10	miR-142-3p^6^, miR-150^7^	*Gfi1*^6^, *Marcks*^6^, *Msh6*^6^, *Pp11r*^7^, *Psmc1*^6,7^
TCell 11	miR-146^8^, miR-16^9^, miR-181b^10^	*Atp1b3*^10^, *Ipo4*^9^, *Klhdc2*^10^, *Mrpl30*^8^, *Orc5l*^8^, *Tuba4*^9^

BCell 3	miR-181b^1^, miR-181c^2^, miR-26a^3^	*Atpif1*^3^, ***Aurkb***^1,2^, *Cbx1*^3^, ***Cdc45l***^2^, ***Cks1b***^1,2^, ***Cks2***^3^, *Cox5a*^3^, *Hmgb2*^1,2^, *Melk*^1,2^, ***Ttk***^1,2^, *Uchl5*^3^
BCell 5	miR-15a^4^, miR-15b^5^, miR-221^6^,	***Cdca4***^4,5^, ***Chek1***^4,5^, ***Mcm4***^7^, *Nasp*^6^, *Nfyb*^6^, ***Smc4l1***^7^
	miR-223^7^	*Tuba2*^4,5,7^
BCell 6	miR-155^8^, miR-191^9^	***Ctps***^9^, *Ddx1*^8^, *Hint1*^9^, ***Mcm2***^8^, *Phf17*^8^, *Prdx4*^9^, *SNrpd1*^9^
BCell 19	miR-142-3p^14^, miR-342^15^	*2410002F23Rik*^14^, *H2-Eb1*^14^, *Ltb*^15^, *Tap2*^14,15^

We display the cluster and data set id, the list of microRNA and list of target genes, with *p*-values *<*0.05 and at least four target genes per cluster. Genes involved in cell proliferation or DNA repair are depicted in bold. The indices indicates to which microRNA a gene is related, when there is more than one enriched microRNA in a cluster.

At the individual gene level we identify some candidate microRNA targets for further detailed analysis: the three known genes (*H2-Eb1*, *Ltb*, *Tap2*) of BCell cluster 19 are all involved in the antigen presentation by MHC class II molecules [33,54]. In the context of the cell cycle, *Chek1 *(clusters TCell 5 and BCell 5) and *Cdc25a *(cluster TCell 5) are important for the transition between G1/S and G2/M phases [55].

Furthermore, both genes are candidate targets of the same microRNA, miR-15a, which is related to apoptosis in chronic lymphoid leukemia cells [56]. Another interesting gene codes for the nuclear factor Y (*Nfyb*; cluster BCell 5), which regulates *Hoxb4 *[57], *Cdc34 *[58] and the major histocompatibility complex in mice [59]. These are all important genes for lymphoid development. The mRNA of the growth factor independence-1 transcription factor (*Gfi1*; cluster TCell 10) is a potential target of miR-142-3p with a function in the restriction of cell proliferation and maintenance of the functional integrity of lymphocyte cells [60]. Moreover, *Gfi1 *is implicated in the transition from CD4/CD8 double negative to double positive T cells [61].

In order to relate our approach with [4,5], we also perform a microRNA enrichment analysis with the results of SOM (see Table S4 and S5 in the Additional data file 3). In TCell there is little overlap between the microRNA targets, with the exception of SOM cluster 6, which is a subset of targets genes from cluster 5 from MixDTrees. We also compare the *p*-values obtained by both methods in a procedure similar to the one performed in [31]. For TCell, MixDTrees results in lower *p*-values in nine out of 14 microRNAs (see Fig. S5 in Additional data file 2). In BCell, gene targets found with SOM are partially a subset of the ones encountered with MixDTrees; 14 out of 24 targets genes in BCell SOM are also detected by MixDTrees (Table S5 in the Additional data file 3). For BCell, (Fig. S6 in Additional data file 2), MixDTrees obtains lower *p*-values in 8 out of 14 microRNAs. Even though SOM obtains lower p-values for microRNAs found to be enriched with both methods, MixDTrees detects seven enriched microRNA not significantly enriched in SOM. An inspection of the cumulative distribution function of these *p*-values also reinforces the view that MixDTrees is more sensitive in detecting enriched microRNAs than SOM in BCell (Fig. S8 in Additional data file 2). Overall, the results suggests a higher sensitivity of MixDTrees-MAP in finding groups of microRNA targets sharing similar expression patterns compared to SOM. Additionally, we performed microRNA enrichment *p*-value comparison between MixDTrees-MAP and MixDTrees-MLE for both data sets (see Additional data file 2 Fig. S9 and S10). For TCell, MixDTrees-MAP achieves a higher enrichment for nine out of 14 microRNAs; while for BCell, six out of 13 microRNAs. In summary, clusters computed according to MAP have an increased enrichment for TCell and a slightly lowered enrichment for BCell. A manual inspection of the contingency table comparing the clusters from MAP and MLE (Additional data file 3 Table S15) and in the cluster size distributions (Additional data file 2 Fig. S11) shows that MixDTrees-MLE has a tendency to produce spurious, small clusters as a result of over-fitting, a known disadvantage of MLE estimates [17]. Note that the resulting *p*-values decrease drastically as a function of the cluster size, making a clustering which joins clusters appear preferable. Enrichment analysis should be used cautiously to compare clusterings, if the cluster size distributions are not similar, as it is the case for the MLE results. This and the results on simulated data supports our preference of MixDTrees-MAP over MixDTrees-MLE.

## Conclusion

The regulatory processes behind cell proliferation and differentiation are of central interest to developmental biologists and clinicians alike and are frequently the focus of large-scale studies to investigate gene expression along paths of differentiation. To make full use of this data in a principled manner we present a novel statistical framework which models gene expression in the course of development. By combining the dependence trees in a classical mixture model, we facilitate interactive querying and visualization of data and, more importantly, the detection of possibly overlapping clusters of co-expressed genes, which provide a basis for the identification of key players in the regulatory mechanism and their mode of action.

In particular, we detect interesting groups of genes not found by classical clustering methods such as SOM. By incorporating microRNA binding data, we show how to identify complex regulatory relationships. Compared to an analysis based only on sequence, we predict a manageable number of plausible microRNA targets. Moreover, our method offers some insights into the biological role of predicted microRNAs, by the inspection of the developmental profiles of gene targets associated with one microRNA. A comparison with SOM indicates that our approach is more sensitive for finding co-expressed genes on which the same microRNA can have a regulatory effect.

Extensions to accommodate further types of data are straightforward. Binding sites of transcription factors can be analyzed completely analogous to the microRNA analysis. If expression levels of microRNAs in developmental stages investigated in TCell or BCell were available, we could incorporate a target prediction framework [62]. Furthermore, we can simply apply established techniques [63-66] to extend our mixture model to integrate heterogeneous data–sequence information, protein interaction, genotype, phenotype data–and semi-supervised extensions to mixture estimation can be applied to make use of biological knowledge about functional similarities and regulatory relationships [22,67,68]. This is of highest relevance, because the identification of regulatory modules is actually feasible compared to the automated inference of regulatory networks [69]. Once a statistical model is obtained, further detailed questions about the significance of differences, or the most likely stage, at which differentiation occurs can be easily answered.

Fascinating extensions are possible, even when one only considers gene expression data and the basic method. None of the currently publicly available data sets offers both a tree with a large number of branches and a detailed view of all, in particular early, development stages ([70] concentrates on mature and immature cells in final development stages); combining data from several microarray platforms suffers from the usual problems. Hence, we concentrate on two smaller but detailed studies covering several stages of T cell and B cell development [4,5], and a tree containing three lineages of lymphoid cells. Note that in the latter several cell types of intermediary development stages are not measured. Nevertheless, our analysis indicates that our method takes advantage of the tree structure information in detecting relevant differences of gene expression in these lineages. This also reinforces the importance of the creation of expression compendia, such as the one in [70], where many intermediary stages of differentiation of the developmental tree are also present. Such data will be of great value as computational methods *can *exploit characteristics intrinsic to cell development.

Lastly, developmental biologists are still redrawing developmental trees with the discovery of new intermediary stages and "alternative" paths of development [1-3]; a particular developmental stage might also be formed by a mixture of distinct cell types not well characterized yet. As an example of an alternative path, there has been evidence that DN1 T cells can be originated not only from the lymphoid progenitor as depicted in Fig. 1, but also from the earlier multipotent progenitor cells [3]. It is an exciting prospect to infer branches and stages of a developmental tree from gene expression data, ideally per functional module. This structure learning (see [16] for discrete data) can be incorporated in the EM-based parameter estimation. In conclusion, our results suggest that the mixture of dependence trees provides a natural and powerful representation of developmental gene expression data. Furthermore, our results reinforce the importance of the creation of detailed and heterogeneous data sets for helping elucidate the regulatory mechanisms of development.

## Methods

### Data

Our work concentrates on two detailed studies covering several stages of the B and T cell development [4,5] and a tree containing three lineages of lymphoid cells [10-12]. All gene expression data sets analyzed are deposited at the Gene Expression Omnibus [71]. Their accession entries are: GDS44 and GDS52 for BCell, GDS237 and GDS257 for TCell, and GDS1077 (HSC), GSE2227 (Bcells) and GDS828 (NK and SP4) for the LymphoidTree data. Final normalized and filtered data sets are found in [26]. Furthermore, we also use simulated data sets in order to evaluate the method. Finally, we describe a set of microRNAs that are used in our study.

#### T cell development (TCell)

This data set contains measurements of gene expression during the development of T cells in mouse [4]. Based on cell surface markers seven stages have been distinguished: CD4 and CD8 double negatives (DN2, DN3, DN4), large double positives (DPL), small double positives (DPS), single positive CD4 (SP4) and single positive CD8 (SP8) (see Fig. 1 for the corresponding tree, and the original publication for details [4]). Affymetrix MU11k chips with four or five replicates were used to measure the expression levels of 13,104 mouse genes. We performed variance stabilization [72] on all chips, and computed the median values of replicates. To facilitate comparisons, we restrict the set to the same list of 1318 differentially expressed genes that was used by Hoffmann and colleagues [4]. Furthermore, we normalize the expression levels separately for each gene to mean zero and standard deviation one, as is routine in gene expression analysis. Finally, we map each probe set to a gene symbol if it exists in the respective chip platform annotation provided by the GEO database [73]. The final dataset is found at Additional data file 4.

#### B cell development (BCell)

This data set contains expression levels of five consecutive stages of the B cell lineage, Pre-BI, large Pre-BII, small Pre-BII, immature, and mature B cells [5]. This study was conducted on Affymetrix MU11k chips also, and we pre-process the data exactly as it is described for TCell. The final dataset is found at Additional data file 5.

#### Lymphoid tree (LymphoidTree)

We combine the data of the wild-type control measurements of three studies [10-12] based on the Affymetrix U74 platform to obtain a development tree with distinct lymphoid lineages. This results in expression values of a hematopoietic stem cell (pHSC) from [10], of Natural Killer cells (NK) and of SP4 cells from [11], and of three B cell stages from [12], which are pro-B, pre-B and immature B cells. We pre-process the data exactly as it is described for TCell. Additionally, we remove genes which do not display a 2-fold change in expression at least once. The final dataset is found at Additional data file 6.

#### Simulated data (SIM)

We use MixDTrees with random parameterizations to generate simulated data. For the tree structure given in Fig. 2, we randomly chose the *μ*_*u*|*v*, *k *_from the range [-1.5, 1.5] and $\sigma_{u|v,k}^{2}$ from [0, 1]. We create five experimental settings to inspect the performance of the method in the presence of distinct levels of dependence. For these five settings, we sample *w*_*u*|*v*, *k *_uniformly from [-*ε*, *ε *] (independent data), [-0.5, 0.5], [-1, 1], [-1.0, -0.5] ∪[0.5, 1] and [-1, -1 + *ε *] ∪ [1 – *ε*, 1] (tree dependent data) respectively, where *ε *= 0.01. We chose *K *= 5 and mixture coefficients equal to *α *= (0.1, 0.15, 0.2, 0.2, 0.35). For each experimental setting, we generate ten such mixtures, and sample 500 development profiles from each (see Additional data file 1 for more results on simulated data and Additional data file 7 for datasets). To evaluate the results we compare the class information from the data generation to compute sensitivity, $\frac{\#TP}{\#TP+\#FN}$, and specificity, $\frac{\#TP}{\#TP+\#FP}$, where, for a given clustering result and the class information, *TP *denotes the number of pairs of objects in the same cluster and same class. The remaining three types of pairs are counted as *FP *(same cluster, distinct class), *TN *(distinct cluster and class) and *FN *(distinct cluster, same class). For each method, we compute the sensitivity and specificity on all 10 data sets of an experimental setting and take the mean (see Fig. 7). To compare MixDTrees-MAP with other methods, we apply a one tailed paired *t*-test to evaluate the hypothesis that two methods have the same mean specificity (or sensitivity) in a given experimental setting. Low *p*-values indicate that the equal means hypothesis was rejected and that mean specificity (or sensitivity) was significantly higher in MixDTrees-MAP. For brevity, in the Simulated data section, we simply state–MixDTrees-MAP had a higher sensitivity than method X (*p*-value below 0.05)–when the null hypothesis is rejected.

#### Lymphoid development related microRNAs (LympMIR)

We collect 17 microRNAs that have been found to be involved in Lymphoid development or at least differentially expressed between distinguishable lymphocyte cell types [7-9,56,74]: mmu-miR-24, mmu-miR-26a, mmu-miR-142-3p, mmu-miR-146, mmu-miR-150, mmu-miR-155, mmu-miR-181a, mmu-miR-181b, mmu-miR-181c, mmu-miR-191, mmu-miR-221, mmu-miR-222, mmu-miR-223 and mmu-miR-342. Additionally, we include mmu-miR-15a, mmu-miR-15b, and mmu-miR-16 because, according to recent papers, they participate in the regulation of cell proliferation and apoptosis [75,76]. Since we refer exclusively to microRNAs of the mouse in this work, the species prefix mmu is omitted throughout the text. The lists of candidate targets of these were obtained in the miRBase Targets database [49] (Version 2.0), which uses the Miranda algorithm [77] to search for possible microRNA binding sites in the gene sequences.

#### Mixtures of dependence trees estimation

We combine *K *DTrees in a mixture $f(x|\Theta )=\sum_{k=1}^{K}\alpha_{k}p[x|\theta_{k}]$, where Θ = (*θ*_1_, ..., *θ*_*K*_, *α*_1_, ..., *α*_*K*_), *θ*_*k *_denotes the parameter set of the *k-*th Dtree and *αk *≥ 0, $\sum_{k=1}^{K}\alpha_{k}=1$, are the mixture weights or component priors. By introducing a discrete hidden variable *Y *= {*y*_*i*_} for 1 ≤ *i *≤ *N*, which indicates which DTree generated which developmental profile *x*_*i*_, we can formulate a complete log-likelihood function and estimate the parameters with the Expectation-Maximization (EM) algorithm [28]. Given an initial parameterization Θ^0^, EM iterates two steps: first estimating the posterior probabilities $P[y_{i}=k|x_{i},\theta_{k}^{m}]$ (E Step), and second the computation of the maximum-likelihood parameters Θ^*m *+ 1 ^(M-step), as defined in Eq. 4, Eq. 5 and Eq. 6. We refer the reader to [36] for details of the EM-algorithm.

To avoid over-fitting the models, in particular for components with low component priors *α*_*k*_–that is, a small number of assigned genes–we propose maximum-a-posteriori (MAP) approach. We assume that *w*_*u*|*v, k *_~ *N*(0, *α*_*k*_*β*_*u*|*v, k*_, $\sigma_{u|k}^{-2}$) [78]. Consequently, the estimates take the form.

$${\hat{w}}_{u|v,k}=\frac{{\hat{\sigma }}_{uv|k}}{{\hat{\sigma }}_{u|k}^{2}(1+\beta_{u|v,k}^{-1})},$$

$${\hat{\sigma }}_{u|v}^{2}={\hat{\sigma }}_{u}^{2}-{\hat{w}}_{u|v}^{2}{\hat{\sigma }}_{v}^{2}(1-\beta_{u|v,k}^{-1}).$$

For the sake of simplicity we omit the coefficients *k *which indicates a tree in a given mixture from formulas in the Dependence tree section. See Protocol for exact MLE and MAP formulas in the mixture context. When *β *→ ∞, we obtain a non-informative prior, for which the MAP and MLE estimates are equal. As *β *→ 0, *w *→ 0 and we have a univariate Gaussian. As in [78], we use a empirical Bayes approach to estimate the value of the hyper-parameter *β*_*u*|*v*, *k *_as

$${\hat{\beta }}_{u|v,k}=\frac{\sum_{i=1}^{N}r_{ik}}{\frac{{\hat{\sigma }}_{u|k}^{2}{\hat{\sigma }}_{v|k}^{2}}{{\hat{\sigma }}_{uv|k}^{2}}-1},$$

where *r*_*ik *_is equal to the posterior probability *P *[*y*_*i *_= *k*|*x*_*i*_, *θ*_*k*_] calculated in the E step. This term can be interpreted as the inverse of the linearity evidence. It penalizes components with low responsibilities and larger variances, enforcing lower *w*_*u*|*v*, *k *_values (see Protocol in Additional data file 1 for derivations of all formulas).

The last step after the mixture estimation is the assignment of genes to groups. This is done by assigning genes to the component that maximizes the posterior of the *i*-th gene, which is *y*_*i *_= *argmax*_1 ≤ *k *≤ *K*_(*r*_*ik*_). Note, that more refined assignment schemes [22] (i.e., decoding a mixture) which increase the robustness of the clustering method can also be used.

### Application in lymphoid development

We perform the following steps on each of the sets TCell, BCell, LymphoidTree, and SIM. The mixture estimation method is initialized with *K *random DTrees, which are obtained by choosing random values uniformly and in [0, 1] independently for each *r*_*ik *_and estimating the individual models. Subsequently, we train the mixture model using the EM-algorithm and MAP estimates. To avoid the effect of the initialization, all estimations are repeated 15 times, and the one with highest likelihoods is selected (a similar procedure is applied for *k*-means and SOM). The implementation of our method (licensed under the GPL) and MS Windows binaries are available at [26]. There you can also find a web database–generated with our MixDTrees Report tool–with results of all analyses described in this article.

On TCell and BCell, we used the SOM results as given by [4,5]. For SOM experiments on SIM data, we used the default parameters of the implementation [25], which uses a set of heuristics to select the values. Furthermore, we performed a clustering of SOM nodes with *k*-means as it is a common practice [79]. In order to facilitate the comparison between our clustering results and the clusters of the original work we reorder our clusters accordingly. Dependence between developmental stages is measured as the correlation between variables. Given two stages, *X*_*u *_and *X*_*v *_the correlation is defined as

$$\rho_{u,v}=\frac{{\hat{\sigma }}_{uv}}{{\hat{\sigma }}_{u}{\hat{\sigma }}_{v}},$$

where -1 ≤ *ρ*_*u*, *v *_≤ 1 and *ρ*_*u*, *v *_= 0 indicates independence of variables.

## Abbreviations

BCell – B cell development data

DTree – dependence tree

DN – CD4-/CD8- double negative cells

DPL – CD4+/CD8+ double positive large cells

DPS – CD4+/CD8+ double positive small cells

FACS – fluorescence activated cell sorting

LympMIR – hematopoiesis related microRNAs data

LymphoidTree – lymphoid tree data

MAP – maximum-a-posteriori

MLE – maximum likelihood estimates (MLE)

MixDTrees – mixtures of dependence trees

MixDTrees-MAP – mixtures of dependence trees with MAP estimates

MixDTrees-MLE – mixtures of dependence trees with MLE estimates

NK – natural killer cells

pHSC – pluri-potent, self-renewing hematopoietic stem cells

SIM – simulated data

SOM – self-organizing maps

SP4 – single positive CD4

SP8 – single positive CD8

TCell – T cell development data

## Competing interests

The author(s) declares that there are no competing interests.

## Authors' contributions

IC implemented the approach and performed the experiments. IC and SR evaluated the results. IC, SR and AS designed this study and wrote the manuscript. All authors read and approved the final manuscript.

## Supplementary Material

Additional data file 1 **Protocol**. This file contains information on software implementations, derivations of estimation formulas and additional experiments with simulated data. Click here for file

Additional data file 2 **Supplementary Figures**. Figures 1, 2 and 3 contains all clusters results from MixDTrees on BCell, TCell and LymphoidTree, and Figure 4 contains BIC results from LymphoidTree. Figures 5 and 6 contain comparisons between microRNA enrichment with MixDTrees-MAP and SOM in TCell and BCell, Figures 7 and 8 depict the empirical cumulative distribution function (cdf) of microRNA enrichment *p*-values from TCell and BCell, and Figures 9 and 10 contain comparisons between microRNA enrichment with MixDTrees-MAP and MixDTrees-MLE in TCell and BCell. Figure 11 describes the cluster size distribution of clustering results in TCell and BCell. Click here for file

Additional data file 3 **Supplementary Tables**. Tables 1, 2 and 3 contains correlation matrices from BCell, TCell and LymphoidTree datasets; Tables 4, 5 and 6 contains enriched microRNA and gene targets from SOM results on TCell and BCell and from MixDTrees-MAP results on LymphoidTree; Tables 7, 8, 9 contains microRNA enrichment *p*-values for BCell, TCell and LymphoidTree on MixDTrees-MAP results; Tables 10 and 11 contains microRNA enrichment *p*-values for BCell and TCell on SOM results; Tables 12 and 13 contain the contingency tables comparing clusters from MixDTrees-MAP and SOM with BCell and TCell datasets; and Tables 14 and 15 contain the contingency tables comparing clusters from MixDTrees-MAP and MixDTrees-MLE with BCell and TCell datasets. Click here for file

Additional data file 4 **TCell Dataset**. Data set after filtering and normalization procedures. The second column indicates the cluster assignment found by the MixDTrees. Click here for file

Additional data file 5 **BCell Dataset**. Data set after filtering and normalization procedures. The second column indicates the cluster assignment found by the MixDTrees. Click here for file

Additional data file 6 **LymphoidTree Dataset**. Data set after filtering and normalization procedures. The second column indicates the cluster assignment found by the MixDTrees. Click here for file

Additional data file 7 **SIM Datasets**. Data sets from simulated MixDTrees. See readme.txt for file descriptions. The first column indicates the true label of the sample. Click here for file
