# Supplementary material for: Gene expression trees in lymphoid development
Source: BMC Immunol. 2007 Oct 9;8:25. doi: 10.1186/1471-2172-8-25 (PMC2244641; doi:10.1186/1471-2172-8-25)
Supplement: Additional data file 1 — Protocol. This file contains information on software implementations, derivations of estimation formulas and additional experiments with simulated data. [file 1471-2172-8-25-S1.pdf]

Protocol - Gene Expression Trees in Lymphoid Development

Ivan G. Costa, Stefan Roepcke, Alexander Schliep
Department of Computational Molecular Biology
Max-Planck-Institute for Molecular Genetics, Berlin, Germany

1 Method Implementation

The Mixture of Dependence Trees `MixDTrees` estimation was implemented in Matlab14 using the Bayesian Network Toolbox (`http://bnt.sourceforge.net/`). All matlab source files, window binaries, tutorials and sample data sets are available at the tool webpage `http://algorithmics.molgen.mpg.de/MixDTrees`. We also used the Bayesian Network Toolbox for implementations of the mixture of Gaussians with full and diagonal matrices. An implementation of the k-means algorithm in Python was obtained at Open Source clustering (`http://bonsai.ims.u-tokyo.ac.jp/~mdehoon/software/cluster/`), while SOM implementation was obtained at SOM Matlab Toolbox (`http://www.cis.hut.fi/projects/somtoolbox/`).

2 Mixture of Dependence Trees Estimation

We present in this section the derivation of the estimates of a `MixDTrees`. We concentrate on the estimates of a mixture of conditional Gaussians, the building blocks of the `MixDTrees`. For more details on mixture models see [?].

2.1 Maximum Likelihood

Consider a continuous bivariate $X = (X_u, X_v)$ and $x = (x_u, x_v)$ a realization of X. For clustering purposes, we also define a unknown discrete variable Y, where the realization $y \in \{1, .., k\}$ indicates the cluster of a given realization x [?]. Setting $\mathrm{pa}(u) = v$ and $y = k$, the conditional Gaussian density function is defined as

$$p[x_u|x_v, y = k, \theta_k] = (\sqrt{2\pi}\sigma_{u|v,k})^{-1} \exp\left(-(x_u - \mu_{u|v,k} - w_{u|v,k}x_v)^2/2\sigma^2_{u|v,k}\right), \tag{1}$$

where $\tau_{uk} = (\sigma_{u|v,k}, \mu_{u|v,k}, w_{u|v,k})$ and $\theta_k = (\tau_{uk}, \tau_{vk})$ are the unknown model parameters.

By marginalizing over y, the hidden variable, we get the following mixture density, or convex combination of component clusters, one component corresponding to one cluster,

$$p[x_u|x_v, \Theta] = \sum_{k=1}^{K} \alpha_k \cdot p[x_u|x_v, y = k, \theta_k],$$

where the mixture weights $\alpha_k = p[y = k|\theta_k]$, $\alpha_k \geq 0$, $\sum_{k=1}^{K} \alpha_k = 1$ and $\Theta = (\theta_1, ..., \theta_K, \alpha_1, .., \alpha_K)$.

Let $x_i = [x_{iu}, x_{iv}]$ be the developmental profile of gene i, and x_{iu} be the expression value of the gene i in development stage u for $1 \leq i \leq N$. The likelihood of the data assuming that genes are independently distributed is given by,

$$p[X_u|X_v, \Theta] = \prod_{i=1}^{N} \sum_{k=1}^{K} \alpha_k \cdot p[x_{iu}|x_{iv}, y_i = k, \theta_k],$$

and the complete likelihood takes the following multinomial form

$$p[X_u, X_v, Y, \Theta] = \prod_{k=1}^{K}\prod_{i=1}^{N}(\alpha_k \cdot p[x_{iu}|x_{iv}, y_i = k, \theta_k])^{r_{ik}},$$

where $\alpha_k = \frac{1}{N}\sum_{i=1}^{N} r_{ik}$ and $r_{ik} = p[y_i = k|x_{iu}, x_{iv}]$ is the posterior probability (or responsibility) [?] that gene i belongs to cluster k.

To simplify computation, we use the log of the complete likelihood CLL in our derivations.

$$\begin{aligned}
\text{CLL} &= \sum_{k=1}^{K}\sum_{i=1}^{N} r_{ik}\left(\alpha_k - \frac{1}{2}\ln(\sigma_{u|v,k}) - \left((x_{iu} - \mu_{u|vk} - w_{u|v,k}x_{iv})^2/2\sigma^2_{u|v,k}\right)\right) \\
&= \sum_{k=1}^{K}\sum_{i=1}^{N} r_{ik}\alpha_k - \frac{1}{2}\sum_{k=1}^{K}\sum_{i=1}^{N} r_{ik}\ln(\sigma_{u|v,k}) \\
&\quad -\sum_{k=1}^{K}\sum_{i=1}^{N} r_{ik}\left((x_{iu} - \mu_{u|v,k} - w_{u|v,k}x_{iv})^2/2\sigma^2_{u|v,k}\right).
\end{aligned}$$

In the following, we find parameters maximizing the logarithm of the complete data likelihood by calculating appropriate derivatives and finding critical points.

2.2 Mean

$$\begin{aligned}
\frac{\partial \text{CLL}}{\partial \mu_{u|k}} &= \sum_{i=1}^{N} r_{ik}\left((x_{iu} - w_{u|v,k}x_{iv} - \mu_{u|v,k})/2\sigma^2_{u|v,k}\right) \\
&= (\sum_{i=1}^{N} r_{ik}x_{iu} - w_{u|v,k}\sum_{i=1}^{N} r_{ik}x_{iv} - \mu_{u|v,k}\sum_{i=1}^{N} r_{ik})/2\sigma^2_{u|v,k}
\end{aligned}$$

Setting to zero, we find,

$$0 = \sum_{i=1}^{N} r_{ik}x_{iu} - w_{u|v,k}\sum_{i=1}^{N} r_{ik}x_{iv} - \mu_{u|v,k} \tag{2}$$

$$\mu_{u|v,k} = \hat{\mu}_{u|k} - \hat{\mu}_{v|k}w_{u|v,k} \tag{3}$$

Note that $\mu_{u|v,k} = \hat{\mu}_{u|k}$, only if $w_{u|v,k} = 0$. For $w_{u|v,k} \neq 0$, $\mu_{u|v,k}$ should be interpreted as the factor of the linear combination. Since this parameter is hard to interpret, we plug in the above definition in the original formula, and adopt the folowing formulation of the conditional Gaussian in the original paper and from here on.

$$p[x_u|x_v, y = k, \theta_k] = (\sqrt{2\pi}\sigma_{u|v,k})^{-1}\exp\left(-(x_u - \mu_{u|k} - w_{u|v,k}(x_v - \mu_{v|k}))^2/2\sigma^2_{u|v,k}\right), \tag{4}$$

2.2.1 Regression Parameter Estimation

For the regression parameter $w_{u|v,k}$ we obtain,

$$\begin{aligned}
\frac{\partial \mathrm{CLL}}{\partial w_{u|v,k}} &= \sum_{i=1}^{N} r_{ik} \left((x_{iu} - \mu_{u|k} - w_{u|v,k}(x_{iv} - \mu_{v|k}))(x_{iv} - \mu_{v|k})/\sigma^2_{u|v,k} \right) \\
&= \sum_{i=1}^{N} r_{ik}(x_{iv}(x_{iu} - \mu_{u|k} - w_{u|v,k}x_{iv} - w_{u|v,k}\mu_{v|k}) \\
&\quad -\mu_{v|k}(x_{iu} - \mu_{u|k} - w_{u|v,k}x_{iv} - w_{u|v,k}\mu_{v|k}))/2\sigma^2_{u|v,k}).
\end{aligned}$$

By definitions of $\hat{\mu}_{u|k}$ and $\hat{\mu}_{v|k}$ this simplifies to,

$$\begin{aligned}
\frac{\partial \mathrm{CLL}}{\partial w_{u|v,k}} &= \left(\sum_{i=1}^{N} r_{ik}x_{iu}x_{iv} - \frac{\sum_{i=1}^{N} r_{ik}x_{iu} \sum_{i=1}^{N} r_{ik}x_{iv}}{\sum_{i=1}^{N} r_{ik}} - w_{u|v,k} \sum_{i=1}^{N} r_{ik}x^2_{iv} \right) /2\sigma^2_{u|v,k} \\
&\quad + \left(\frac{w_{u|v,k} \sum_{i=1}^{N} r_{ik}x_{iv} \sum_{i=1}^{N} r_{ik}x_{iv}}{\sum_{i=1}^{N} r_{ik}} \right) /2\sigma^2_{u|v,k}.
\end{aligned}$$

Setting to zero, we find,

$$\begin{aligned}
0 &= \sum_{i=1}^{N} r_{ik}x_{iu}x_{iv} - w_{u|v,k} \sum_{i=1}^{N} r_{ik}x^2_{iv} - \frac{\sum_{i=1}^{N} r_{ik}x_{iu} \sum_{i=1}^{N} r_{ik}x_{iv}}{\sum_{i=1}^{N} r_{ik}} + \frac{w_{u|v,k} \sum_{i=1}^{N} r_{ik}x_{iv} \sum_{i=1}^{N} r_{ik}x_{iv}}{\sum_{i=1}^{N} r_{ik}} \\
&= \mathrm{E}[x_u, x_v]_k - \hat{\mu}_{u|k}\hat{\mu}_{v|k} - w_{u|v,k}(\mathrm{E}[x^2_v]_k - \hat{\mu}_{v|k}\hat{\mu}_{v|k}) \\
&= \hat{\sigma}_{u,v|k} - w_{u|v,k}\hat{\sigma}^2_{v|k}
\end{aligned}$$

,
and hence,

$$\hat{w}_{u|v,k} = \frac{\hat{\sigma}_{u,v|k}}{\hat{\sigma}^2_{v|k}}. \quad (5)$$

2.2.2 Variance

To simplify the derivations for the variance parameter we substitute $(\sigma^2_{u|v,k})^{-1}$ by $\gamma_{u|v,k}$ and obtain,

$$\mathrm{CLL} = \sum_{k=1}^{K} \sum_{i=1}^{N} r_{ik} \ln(\gamma^{1/2}_{u|v,k}) - \sum_{k=1}^{K} \sum_{i=1}^{N} r_{ik}(x_{iu} - \mu_{u|k} - w_{u|v,k}(x_{iv} - \mu_{v|k}))^2 \gamma_{u|v,k}/2).$$

$$\frac{\partial \mathrm{CLL}}{\partial \gamma_{u|v,k}} = -\frac{1}{2} \sum_{i=1}^{N} r_{ik}(\gamma_{u|v,k})^{-1} - \frac{1}{2} \sum_{i=1}^{N} r_{ik}(x_{iu} - \mu_{u|k} - w_{u|v,k}(x_{iv} - \mu_{v|k}))^2.$$

Setting to zero we have

$$(\gamma_{u|v,k})^{-1} = \frac{\sum_{i=1}^{N} r_{ik}(x_{iu} - \mu_{u|k} - w_{u|v,k}(x_{iv} - \mu_{v|k}))^2}{\sum_{i=1}^{N} r_{ik}}.$$

By definition of $\hat{\sigma}^2_{u|k}$, $\hat{\sigma}^2_{v|k}$ and $\hat{\sigma}_{y,x|k}$ we arrive at

$$
\begin{aligned}
(\gamma_{u|v,k})^{-1} &= \frac{\sum_{i=1}^N r_{ik}(x_{iu}-\mu_{u|k})^2 - 2\sum_{i=1}^N r_{ik}w_{u|v,k}(x_{iu}-\mu_{u|k})(x_{iv}-\mu_{v|k}) + \sum_{i=1}^N r_{ik}w^2_{u|v,k}(x_{iv}-\mu_{v|k})^2}{\sum_{i=1}^N r_{ik}} \\
&= \hat{\sigma}^2_{u|k} - 2w_{u|v,k}\hat{\sigma}_{u,v|k} - w^2_{u|v,k}\hat{\sigma}^2_{v|k}.
\end{aligned}
$$

By definition of w, this yields,

$$
(\gamma_{u|v,k})^{-1} = \sigma^2_{u|v,k} = \hat{\sigma}^2_{u|k} - w^2_{u|v,k}\hat{\sigma}^2_{v|k}. \tag{6}
$$

2.2.3 Sufficient statistics

$$
\hat{\mu}_{v|k} = \frac{\sum_{i=1}^N r_{ik}x_{iv}}{\sum_{i=1}^N r_{ik}} \tag{7}
$$

$$
\hat{\mu}_{u|k} = \frac{\sum_{i=1}^N r_{ik}x_{iu}}{\sum_{i=1}^N r_{ik}} \tag{8}
$$

$$
\hat{\sigma}^2_{v|k} = \frac{\sum_{i=1}^N r_{ik}(x_{iv}-\hat{\mu}_{v|k})^2}{\sum_{i=1}^N r_{ik}} \tag{9}
$$

$$
\hat{\sigma}^2_{u|k} = \frac{\sum_{i=1}^N r_{ik}(x_{iu}-\hat{\mu}_{u|k})^2}{\sum_{i=1}^N r_{ik}} \tag{10}
$$

$$
\hat{\sigma}_{u,v|k} = \frac{\sum_{i=1}^N r_{ik}(x_{iv}-\hat{\mu}_{v|k})(x_{iu}-\hat{\mu}_{u|k})^2}{\sum_{i=1}^N r_{ik}} \tag{11}
$$

2.3 Maximum a Priori

We propose a Maximum a Priori (MAP) estimation to regularize the parameter $w_{y|x,k}$ and avoid over fitting when there is low evidence for a given model (or low α_k).

2.3.1 Prior on $w_{y|x,k}$

We define the prior of $w_{y|x,k}$ to be proportional to

$$
p[w_{u|v,k}] \sim N(0, \frac{\beta_{u|v,k}}{\sum_{i=1}^N r_{ik}}) = (\frac{\sum_{i=1}^N r_{ik}}{\sqrt{2\pi}\beta_{u|v,k}})\exp(-\frac{w^2_{u|v,k}\sum_{i=1}^N r_{ik}}{\beta_{u|v,k}}), \tag{12}
$$

where $\beta_{u|v,k}$ is a hyper-parameter.
The distribution $P[w_{u|v,k}] \sim N(0, \beta_{u|v,k}\sigma^2_{u|v,k}(\sum_{i=1}^N r_{ik}\sigma^2_{v|k})^{-1})$, which is invariant to the scale of the variates X_u and X_v, is a more appropriate extension of the above.

The MAP is obtained from the posterior

$$
p[\Theta|X_u, X_v, Y] = p[X_u, X_v, Y|\Theta]\prod_{k=1}^K P[w_{u|v,k}],
$$

yielding

$$\begin{aligned}\text{MAP} &= -\frac{1}{2}\sum_{k=1}^{K}\sum_{i=1}^{N} r_{ik}\ln(\sigma_{u|v,k}) - \sum_{k=1}^{K}\sum_{i=1}^{N} r_{ik}\left((x_{iu}-\mu_{u|k}-w_{u|v,k}(x_{iv}-\mu_{v|k}))^2/2\sigma^2_{u|v,k}\right)\\ &\quad -\frac{1}{2}\sum_{k=1}^{K}\ln\left(\frac{\beta_{u|v,k}\sigma^2_{u|v,k}}{\sigma^2_{v|k}\sum_{i=1}^{N} r_{ik}}\right) - \sum_{k=1}^{K}\frac{w^2_{u|v,k}\sigma^2_{v|k}\sum_{i=1}^{N} r_{ik}}{\beta_{u|v,k}\sigma^2_{u|v,k}}.\end{aligned}$$

We can take the derivate MAP with respect to $w_{u|v,k}$ as follows

$$\frac{\partial \text{MAP}}{\partial w_{u|v,k}} = \sum_{i=1}^{N} r_{ik}\left((x_{iu}-\mu_{u|k}-w_{u|v,k}(x_{iv}-\mu_{v|k}))x_{iv}/\sigma^2_{u|v,k}\right) - \frac{w_{u|v,k}\sum_{i=1}^{N} r_{ik}\sigma^2_{v|k}}{\beta_{u|v,k}\sigma^2_{u|v,k}},$$

$$0 = \sigma_{u,v|k} - w_{u|v,k}\sigma^2_{v|k} - \frac{w_{u|v,k}\hat{\sigma}^2_{v|k}}{\beta_{u|v,k}},$$

and obtain the map estimate,

$$\hat{w}_{u|v,k} = \frac{\hat{\sigma}_{u,v|k}}{\hat{\sigma}^2_{v|k}(1+\beta^{-1}_{u|v,k})}. \tag{13}$$

When $\beta_{u|v,k} \to \infty$, the prior becomes non-informative; that is, the MAP and ML estimates are equal.
The MAP estimator of $\sigma^2_{u|v,k}$ can be derived as (again we substitute $(\sigma^2_{u|v,k})^{-1}$ by $\gamma_{u|v,k}$) :

$$\frac{\partial \text{MAP}}{\partial \gamma_{u|v,k}} = -\frac{1}{2}\sum_{i=1}^{N} r_{ik}(\gamma_{u|v,k})^{-1} - \frac{1}{2}\sum_{i=1}^{N} r_{ik}(x_{iu}-\mu_{u|k}-w_{u|v,k}(x_{iv}-\mu_{v|k}))^2 - \frac{w^2_{u|v,k}\sum_{i=1}^{N} r_{ik}\sigma^2_{v|k}}{\beta_{u|v,k}},$$

$$0 = -(\gamma_{u|v,k})^{-1} + \sigma^2_{u|k} - w^2_{u|v,k}\sigma^2_{v|k} - \frac{w^2_{u|v,k}\sigma^2_{v|k}}{\beta_{u|v,k}},$$

$$(\gamma_{u|v,k})^{-1} = \hat{\sigma}^2_{u|v,k} = \sigma^2_{u|k} - w^2_{u|v,k}\sigma^2_{v|k}(1+\beta^{-1}_{u|v,k}). \tag{14}$$

Again, when $\beta_{u|v,k} \to \infty$, the prior becomes non-informative.
In a empirical Bayes approach [2], we can estimate the maximum a posteriori value of $\beta_{u|v,k}$ from the data,

$$\frac{\partial \text{MAP}}{\partial \beta_{u|v,k}} = \frac{1}{2\beta_{u|v,k}} - \frac{w^2_{u|v,k}\sum_{i=1}^{N} r_{ik}\sigma^2_{v|k}}{2\beta_k^2\sigma^2_{u|v,k}},$$

and hence

$$\beta_{u|v,k} = \frac{\sigma^2_{v|k}\sum_{i=1}^{N} r_{ik}w^2_{u|v,k}}{\sigma^2_{u|v,k}} = \frac{\sigma^2_{u,v|k}\sum_{i=1}^{N} r_{ik}}{2\sigma^2_{v|k}\sigma^2_{u|v,k}}$$

Since the MAP estimates of $\sigma^2_{u|v,k}$ and $w_{u|v,k}$ are also dependent on $\beta_{u|v,k}$, we approximate the estimate with the MLE definitions of $\hat{\sigma}^2_{u|v,k}$ and $\hat{w}_{u|v,k}$; this yields:

$$\hat{\beta}_{u|v,k} = \frac{\sum_{i=1}^{N} r_{ik}}{\frac{\sigma^2_{u|k}\sigma^2_{v|k}}{\sigma^2_{u,v|k}} - 1}. \tag{15}$$

This empirical prior penalizes variables with large variances (and low covariances) enforcing lower $w_{u|v,k}$, thus avoiding over-fitting.

Figure 1: We display the tree topology used in the additional simulated data sets.

Figure 2: Specificity and Sensitivity vs. noise ratio in the simulated data with equal size components. The noise ratio is measured by dividing the added uniform noise by the mean $\sigma^2_{u|v,k}$ of the original tree.

3 Results

The normalized data sets of `Tcell`, `Bcell` and `LymphoidTree` can be found at `http://algorithmics.molgen.mpg.de/MixDTrees/Data`. The first column corresponds to the gene identifier and the second column to the cluster id. The Mixture of Dependence Trees Reports from the data set analysis are available at `http://algorithmics.molgen.mpg.de/MixDTrees/Reports`.

3.1 Additional Simulated Results SIM2

We use `MixDTrees` with random parameterizations to generate simulated data. For the tree structure given in Fig.1, we randomly chose the $\mu_{u|v,k}$ from the range $[-1.5, 1.5]$, $\sigma^2_{u|v,k}$ from $[0, 0.5]$ and $w_{u|v,k}$ from $[-0.5, 0.5]$. We performed experiments with $k = 3, 5, 10$ and 14 in three component distributions settings: uniform, one big component ($\alpha = 0.5$) and two small components ($\alpha = 0.05$). For each setting, we generated one mixture, and sampled 1000 development profiles.

To inspect the robustness of the method, we added normally distributed noise with mean zero and variance in the interval $[0.0, 1.0]$. In all three experimental settings (Fig.1,Fig.2 and Fig.3), sensitivity and specificity do not decrease significantly until large amount of noise is present ($\sigma_{noise}/\sigma_{sample} > 1$).

References

[1] McLachlan G, Peel D (2000) Finite Mixture Models. Wiley Series in Probability and Statistics. Wiley, New York.

[2] Minka TP (2001) Bayesian linear regression. Technical report, MIT.

Figure 3: Specificity and Sensitivity vs. noise ratio in the simulated data with one large component. The noise ratio is measured by dividing the added uniform noise by the mean $\sigma^2_{u|v,k}$ of the original tree.

Figure 4: Specificity and Sensitivity vs. noise ratio in the simulated data with two small components. The noise ratio is measured by dividing the added uniform noise by the mean $\sigma^2_{u|v,k}$ of the original tree.
