# Supplementary material for: Gene expression trees in lymphoid development
Source: BMC Immunol. 2007 Oct 9;8:25. doi: 10.1186/1471-2172-8-25 (PMC2244641; doi:10.1186/1471-2172-8-25)
Supplement: Additional data file 2 — Supplementary Figures. Figures 1, 2 and 3 contains all clusters results from MixDTrees on BCell, TCell and LymphoidTree, and Figure 4 contains BIC results from LymphoidTree. Figures 5 and 6 contain comparisons between microRNA enrichment with MixDTrees-MAP and SOM in TCell and BCell, Figures 7 and 8 depict the empirical cumulative distribution function (cdf) of microRNA enrichment p-values from TCell and BCell, and Figures 9 and 10 contain comparisons between microRNA enrichment with MixDTrees-MAP and MixDTrees-MLE in TCell and BCell. Figure 11 describes the cluster size distribution of clustering results in TCell and BCell. [file 1471-2172-8-25-S2.pdf]

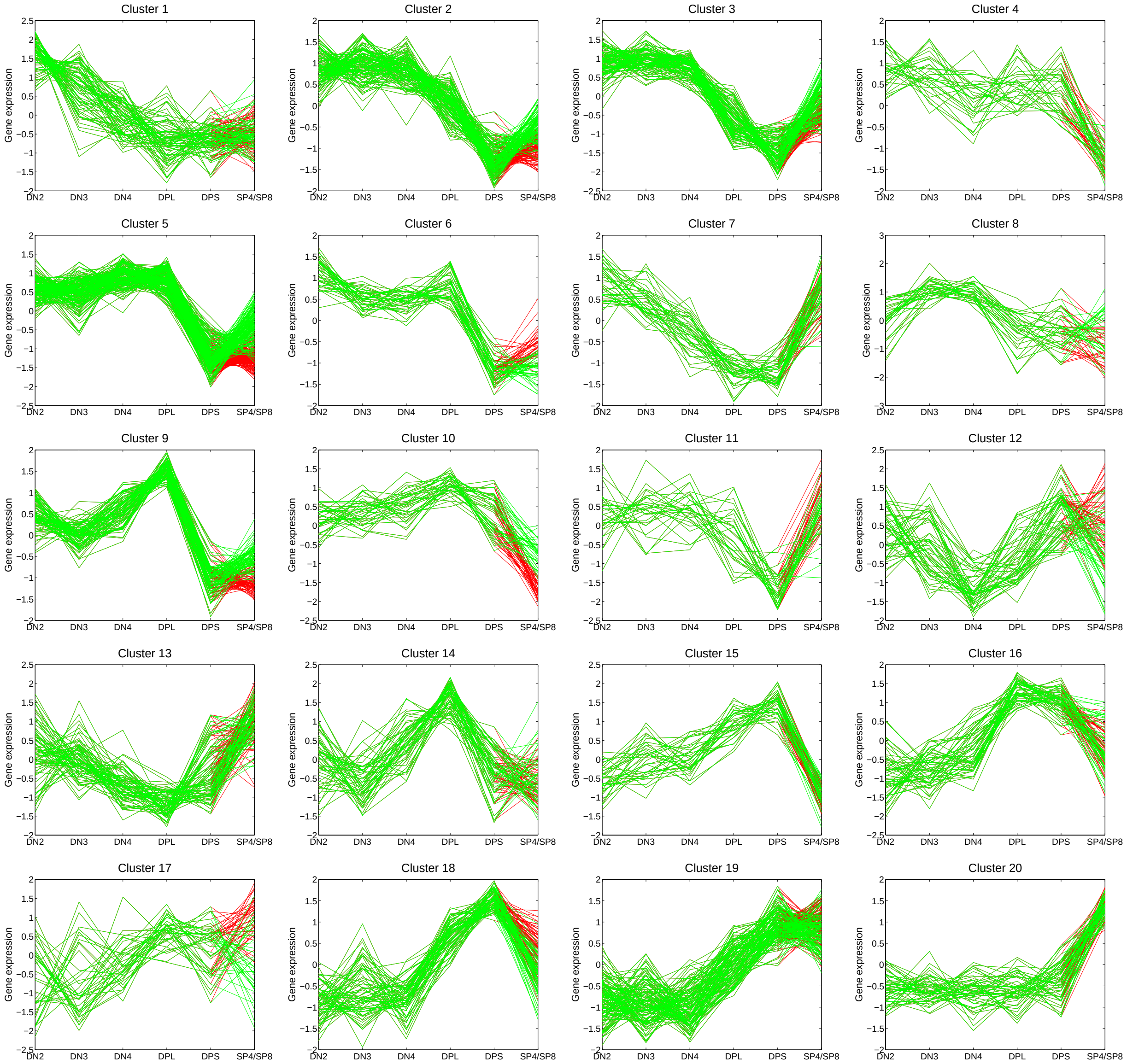

Figure S 1: Clusters from MixDTrees-MAP on TCell. We depict the 20 clusters found in TCell, expression values on the y-axis, and cell types on the x-axis. Lines corresponding to developmental profile values between stages DN2, DN3, DN4, DPL, DPS and SP4 are in green and between DPS and SP8 in red.

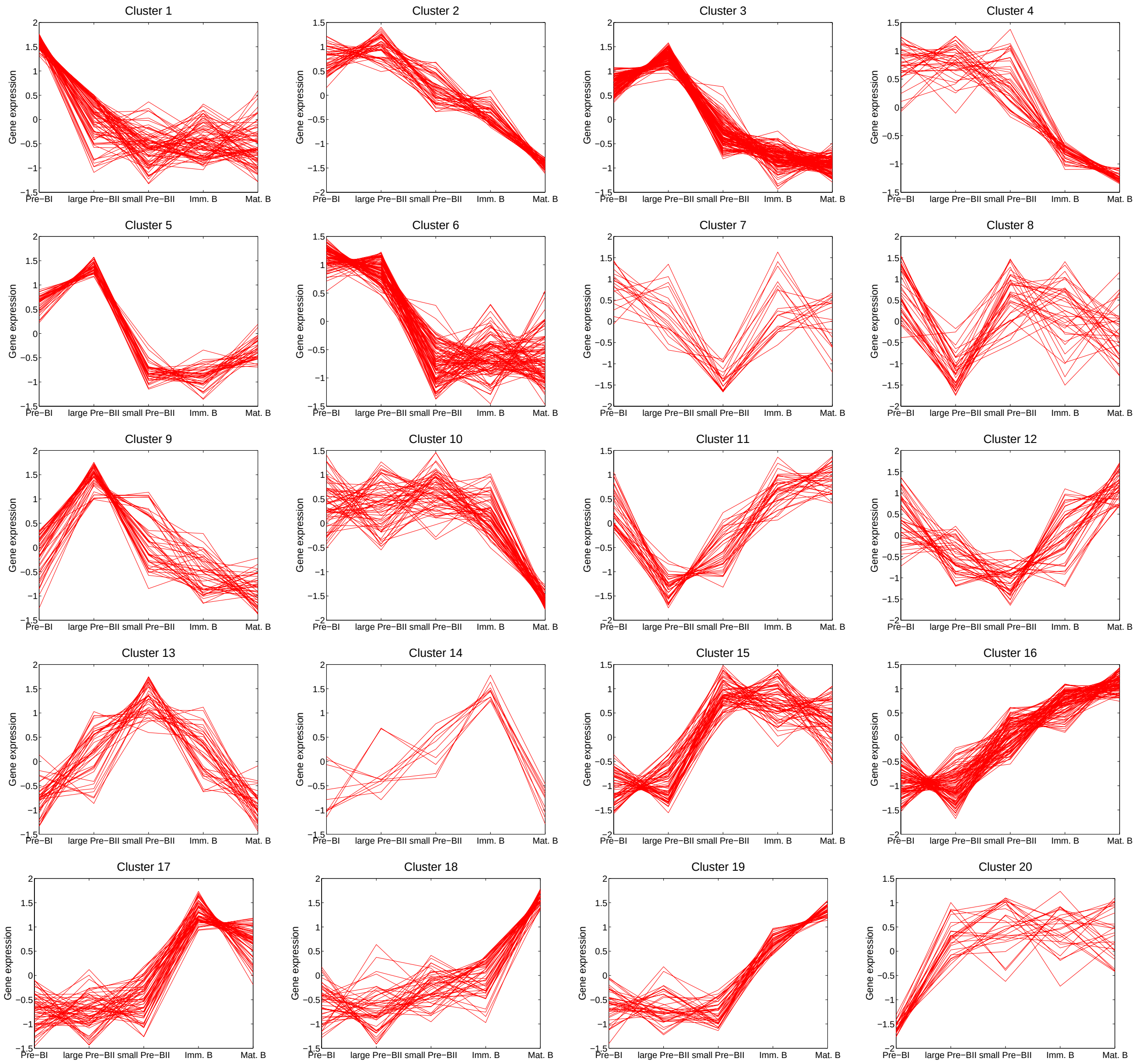

Figure S 2: Clusters from `MixDTrees-MAP` on `BCell`. We depict the 20 clusters found in `BCell`, expression values on the y-axis, and cell types on the x-axis. Lines corresponding to developmental profile values between between all stages are in red.

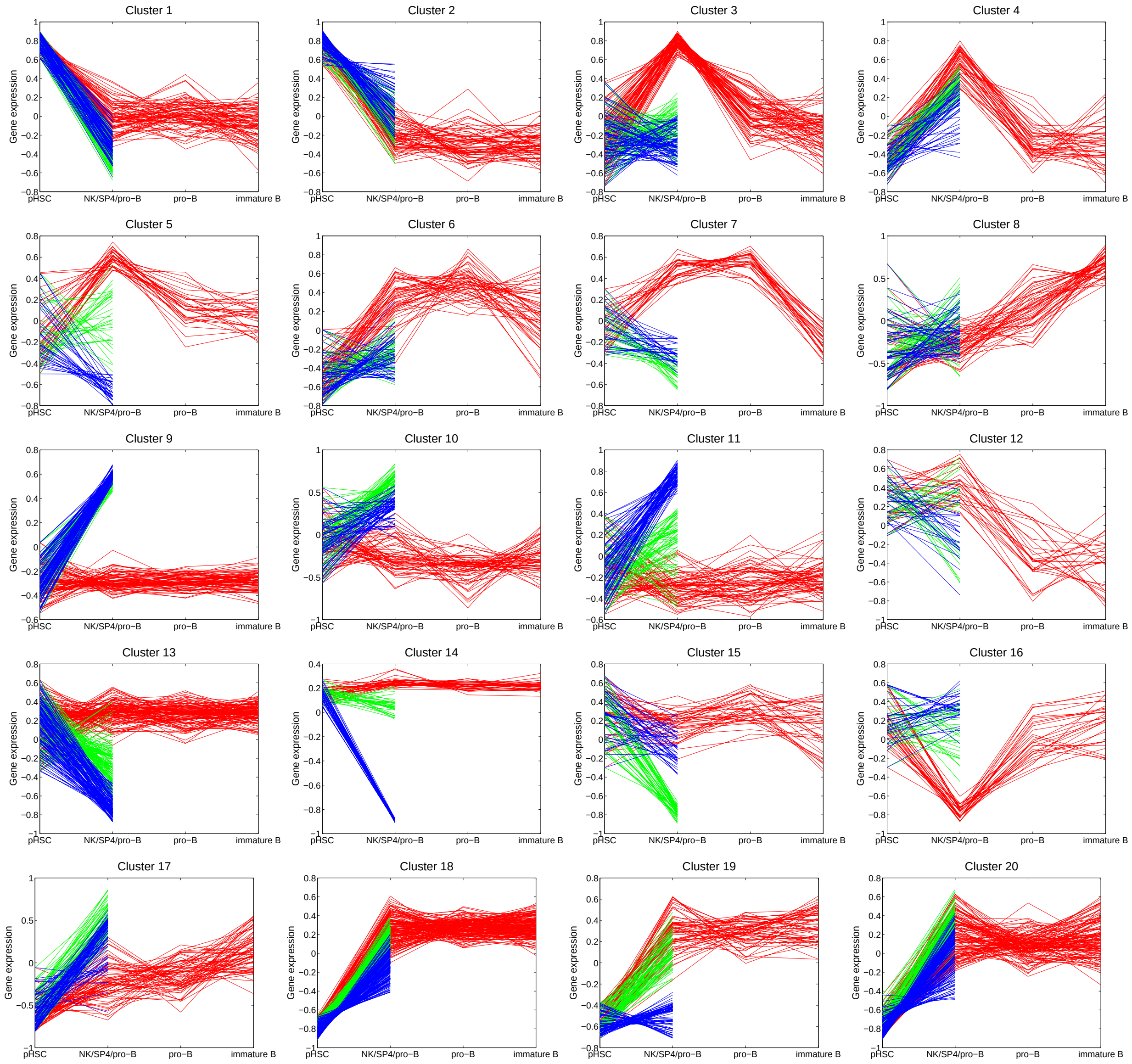

Figure S 3: Clusters from `MixDTrees-MAP` on `LymphoidTree`. We depict the 20 clusters found in `LymphoidTree`, expression values on the y-axis, and cell types on the x-axis. Lines corresponding to developmental profile values between stages HSC, pro-B, pre-B and immature B Cell are in read, between HSC and Natural Killer in blue, and between HSC and SP4 in green.

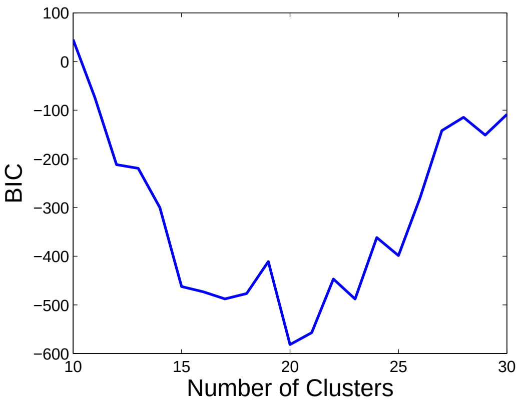

Figure S 4: BIC vs number of components in the data set `LympTree` from `MixDTrees-MAP`. The minimum BIC value obtained indicates the 'optimal' number of clusters, 20 for this data set.

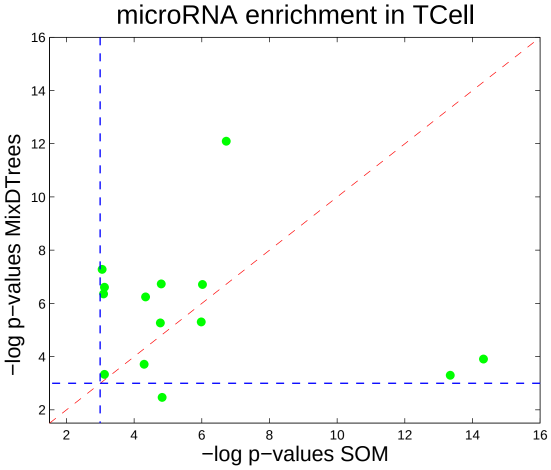

Figure S 5: Scatter plot comparing the microRNAs enrichment of `SOM` (x-axis) and `MixDTrees-MAP` (y-axis) on `TCell`. For visual purposes, we use $-log(p)$-values, where higher values indicates a higher enrichment. The blue lines corresponds to $-log(p)$-value cut-off used (p-value of 0.05). Values to the left of the vertical blue line (below the horizontal blue line) indicate that no enrichment was found for `MixDTrees-MAP` (or `SOM`). We included only microRNAs with a $-log(p)$-value higher then (2.99) in one of the results. Points above the red line indicates a higher enrichment for `MixDTrees-MAP` results, and values bellow it to `SOM`. `MixDTrees-MAP` has a higher $-log(p)$-value in 9 out of the 14 microRNAs, while SOM has enrichment for one microRNA, which is not detected in `MixDTrees-MAP`.

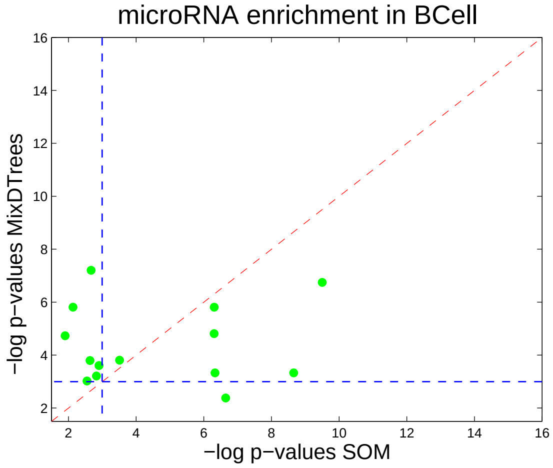

Figure S 6: Scatter plot comparing the microRNA enrichment of `SOM` (x-axis) and `MixDTrees-MAP` (y-axis) on `BCell`. For visual purposes, we use $-log(p)$-values, where higher values indicates a higher enrichment. The blue lines corresponds to $-log(p)$-value cut-off used (p-value of 0.05). Values to the left of the vertical blue line (below the horizontal blue line) indicate that no enrichment was found for `MixDTrees-MAP` (or `SOM`). We included only microRNAs with a $-log(p)$-value higher then (2.99) in one of the results. Points above the red line indicate a higher enrichment for `MixDTrees-MAP`, and values bellow it to `SOM`. `MixDTrees-MAP` has a higher $-log(p)$-value in 8 out of the 14 microRNAs, and it also has enrichment in 7 microRNAs not find with `SOM`.

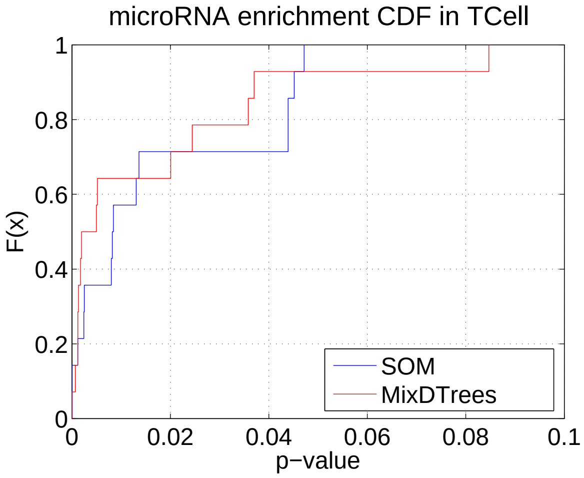

Figure S 7: Empirical cumulative distribution function (CDF) of microRNAs enrichment of `SOM` (blue line) and `MixDTrees-MAP` (red line) on `TCell`. The plots shows no clear distinctions between the CDFs of the two methods.

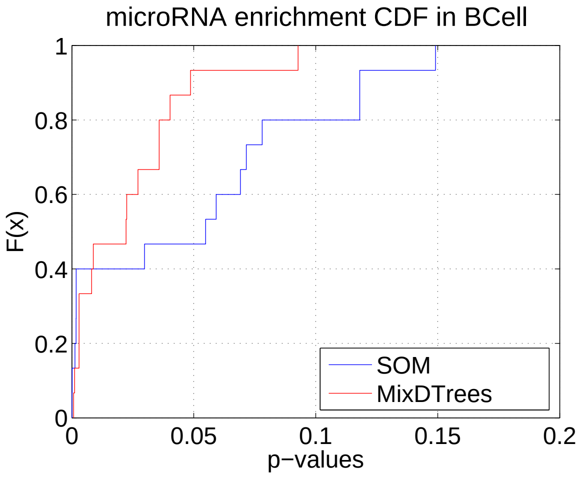

Figure S 8: Empirical cumulative distribution function (CDF) comparing the microRNAs enrichment of `SOM` (blue line) and `MixDTrees-MAP` (red line) on `BCell`. `MixDTrees-MAP` has clearly a p-value distribution towards lower values than `SOM`.

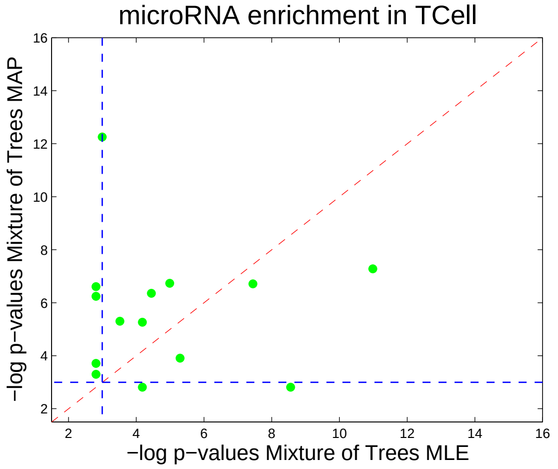

Figure S 9: Scatter plot comparing the microRNA enrichment of `MixDTrees-MLE` (x-axis) and `MixDTrees-MAP` (y-axis) on `TCell`. For visual purposes, we use $-log(p)$-values, where higher values indicates a higher enrichment. The blue lines corresponds to $-log(p)$-value cut-off used (p-value of 0.05). Values to the left of the vertical blue line (below the horizontal blue line) indicate that no enrichment was found for `MixDTrees-MAP` (or `MixDTrees-MLE`). We included only microRNAs with a $-log(p)$-value higher then (2.99) in one of the results. Points above the red line indicate a higher enrichment for `MixDTrees-MAP`, and values bellow it to `MixDTrees-MLE`. `MixDTrees-MAP` has a higher $-log(p)$-value in 9 out of the 14 microRNAs, and it also has enrichment in 4 microRNAs not enriched with `MixDTrees-MLE`.

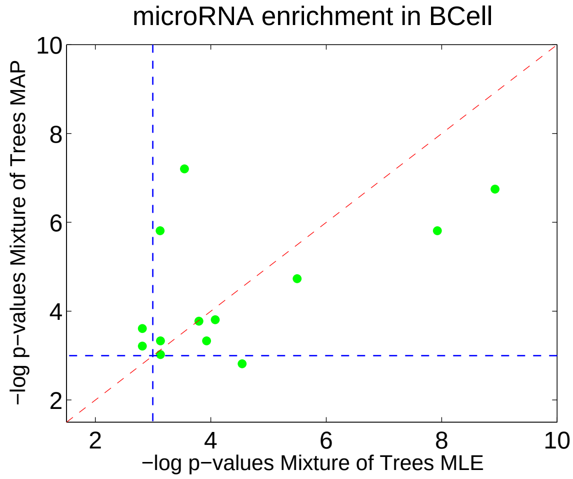

Figure S 10: Scatter plot comparing the microRNA enrichment of `MixDTrees-MLE` (x-axis) and `MixDTrees-MAP` (y-axis) on `BCell`. For visual purposes, we use $-log(p)$-values, where higher values indicates a higher enrichment. The blue lines corresponds to $-log(p)$-value cut-off used (p-value of 0.05). Values to the left of the vertical blue line (below the horizontal blue line) indicate that no enrichment was found for `MixDTrees-MAP` (or `MixDTrees-MLE`). We included only microRNAs with a $-log(p)$-value higher then (2.99) in one of the results. Points above the red line indicate a higher enrichment for `MixDTrees-MAP`, and values bellow it to `MixDTrees-MLE`. `MixDTrees-MAP` has a higher $-log(p)$-value in 6 out of the 13 microRNAs, and it also has enrichment in 2 microRNAs not enriched with `MixDTrees-MLE`.

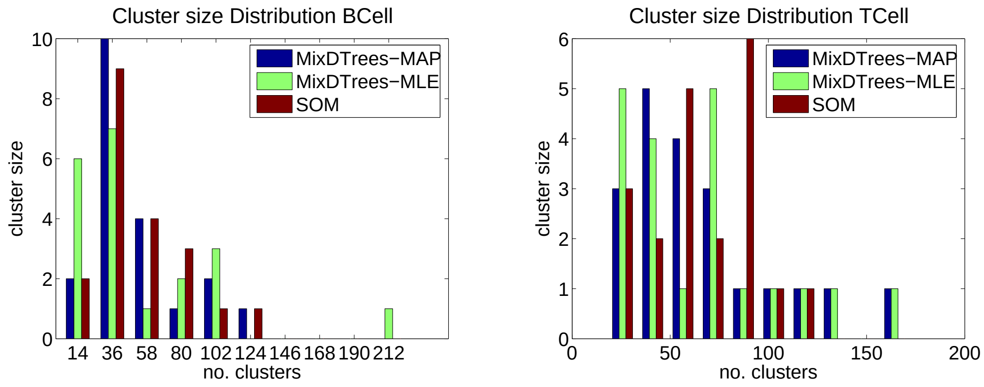

Figure S 11: Cluster size distributions for `MixDTree-MAP`, `MixDTree-MLE` and `SOM` results on `BCell` (left) and `TCell` (right). For `BCell`, `SOM` and `MixDTrees-MAP` have similar cluster size distribution, while `MixDTrees-MLE` has more clusters of small size (6 with less then 25 genes) and one with large size (more then 200 genes). For `TCell`, cluster size distributions do not differ, however, `MixDTrees-MLE` has more smaller clusters, 5 with less than 26 genes.
