# Supplementary material for: Gene expression trees in lymphoid development
Source: BMC Immunol. 2007 Oct 9;8:25. doi: 10.1186/1471-2172-8-25 (PMC2244641; doi:10.1186/1471-2172-8-25)
Supplement: Additional data file 3 — Supplementary Tables. Tables 1, 2 and 3 contains correlation matrices from BCell, TCell and LymphoidTree datasets; Tables 4, 5 and 6 contains enriched microRNA and gene targets from SOM results on TCell and BCell and from MixDTrees-MAP results on LymphoidTree; Tables 7, 8, 9 contains microRNA enrichment p-values for BCell, TCell and LymphoidTree on MixDTrees-MAP results; Tables 10 and 11 contains microRNA enrichment p-values for BCell and TCell on SOM results; Tables 12 and 13 contain the contingency tables comparing clusters from MixDTrees-MAP and SOM with BCell and TCell datasets; and Tables 14 and 15 contain the contingency tables comparing clusters from MixDTrees-MAP and MixDTrees-MLE with BCell and TCell datasets. [file 1471-2172-8-25-S3.pdf]

Table S 1: Correlation matrix of TCell.

	DN2	DN3	DN4	DPL	DPS	SP4	SP8
DN2	1.0000	0.4442	0.3730	-0.2087	-0.6127	-0.4241	-0.3302
DN3	0.4442	1.0000	0.4862	-0.2735	-0.5346	-0.4996	-0.3324
DN4	0.3730	0.4862	1.0000	0.2107	-0.6274	-0.6943	-0.4541
DPL	-0.2087	-0.2735	0.2107	1.0000	0.0914	-0.4505	-0.4355
DPS	-0.6127	-0.5346	-0.6274	0.0914	1.0000	0.3066	0.0019
SP4	-0.4241	-0.4996	-0.6943	-0.4505	0.3066	1.0000	0.5468
SP8	-0.3302	-0.3324	-0.4541	-0.4355	0.0019	0.5468	1.0000

Table S 2: Correlation matrix of BCell.

	Pre-BI	Pre-BII Large	Pre-BII Small	Imm. B-cell	Mature B-Cell
Pre-BI	1.0000	0.5682	-0.3058	-0.7335	-0.6415
Pre-BII Large	0.5682	1.0000	-0.1152	-0.7883	-0.7622
Pre-BII Small	-0.3058	-0.1152	1.0000	0.0331	-0.2941
Imm. B-cell	-0.7335	-0.7883	0.0331	1.0000	0.6001
Mature B-Cell	-0.6415	-0.7622	-0.2941	0.6001	1.0000

Table S 3: Correlation matrix of LymphoidTree.

	HSC	Pro-B	Pre-B	Imat. B	SP4 TCell	NK
HSC	1.0000	-0.2184	-0.2467	-0.3533	-0.5077	-0.1692
Pro-B	-0.2184	1.0000	0.3315	-0.0142	-0.2622	-0.5725
Pre-B	-0.2467	0.3315	1.0000	0.4930	-0.5040	-0.5759
Imat. B	-0.3533	-0.0142	0.4930	1.0000	-0.1911	-0.4381
SP4 TCell	-0.5077	-0.2622	-0.5040	-0.1911	1.0000	0.4525
NK	-0.1692	-0.5725	-0.5759	-0.4381	0.4525	1.0000

Table S 4: List of LympMIR enriched on the clusters of TCell obtained with SOM. We display the cluster id, the list of microRNA and list of target genes, with p-values < 0.05 and at least four target genes. The indices indicates to which microRNA a gene is related, when there is more than one enriched microRNA in a cluster. There is little overlap between the set of target genes below and the ones detected with the MixDTrees. An exception is cluster SOM 6, which has a subset of six genes from cluster TCell 5 (Table 1). In relation to specific genes, cluster SOM 16 contains H2-Q7 (H-2 class I histocompatibility antigen, Q7 alpha chain precursor) and Il7r (Interleukin 7 Receptor-alpha), but it misses some of the interesting target genes discussed in Section 3 of the manuscript.

ID	MicroRNA	Target Genes
SOM 6	miR-15a[1],miR-15b[2], miR-221[3],miR-26a[4]	Atpif1[1,2,4], **Cdc25a**[1,2], **Chek1**[1,2], Cul2[1,2], Kpnb1[3], **Mcm5**[3], Nasp[3,4], Uchl5[4]
SOM 15	miR-342	Arhgdib, Ctsb, D19Wsu162e, Ltb, Rab3ip
SOM 16	miR-16[5],miR-181a[6], miR-181b[7],miR-24[8]	Acvrl1[8], Atp1b3[6,7], Edg1[6,7,8], Gimap4[5], H2-Q7[6,7], Il7r[5], Itm2a[6,7], Phyh[8], Rgl2[8], Tuba4[5], Tyki[8]
SOM 20	miR-146[9],miR-222[10]	Mrpl30[9], Nme1[10], Orc5l[9], Rcn1[10]

Table S 5: List of LympMIR enriched on the clusters of BCell obtained with SOM. We display the cluster id, the list of microRNA and list of target genes , with p-values < 0.05 and at least 4 target genes. The indices indicates to which microRNA a gene is related, when there is more than one enriched microRNA in a cluster. Out of the 24 target genes, 14 were also detected with the MixDTrees (Table 1). This is not a surprise, since these clusters (SOM 4, SOM 6 and SOM 7) correspond to reassigments of genes from clusters 3, 5 and 6 obtained with MixDTrees (see Table S 13). However, SOM results did not detected any of the genes of cluster BCell 19, which contains several interesting targets involved in antigen presentation. In other words, the set of enriched microRNA and gene targets in SOM results represent a subset of the ones encountered with the MixDTrees

ID	MicroRNA	Target Genes
SOM 4	miR-26a	Atpif1, Cox5a, Hmga1, Psmd14
SOM 6	miR-155[1],miR-221[2],miR-223[3]	Arl1[3], Cbx1[3], Fus[3], **Mcm2**[1], **Mcm4**[3],**Mcm5**[2], Mki67[2], Nasp[2], Phf17[1], Ranbp1[3], Stmn1[2,3], Trip13[1]
SOM 7	miR-181a[4],miR-181b[5],miR-181c[6]	**Aurkb**[4,5,6], **Cdc45l**[6], **Cks1b**[4,5,6], Hmgb2[4,5,6], Melk[4,5,6], **Rad21**[4,5], **Smc4l1**[4,5,6], Ttk[4,5,6]

Table S 6: List of `LympMIR` enriched on the clusters of `LymphoidTree` obtained with `MixDTrees`. We display the cluster id, the list of microRNA and list of target genes , with p-values < 0.05 and at least 4 target genes. The indices indicates to which microRNA a gene is related, when there is more than one enriched microRNA in a cluster.

ID	MicroRNA	Target Genes
4	miR-26a[1],miR-342[2]	Epha2[1], Pik3cd[2], Pim1[1], Sec23a[1,2]
6	miR-150[5],miR-191[6]	Arntl[6], Kifc1[5], Pttg1[6], Tubb5[5,6]
11	miR-181a[7],miR-181b[8],miR-181c[9]	Gzma[7,8,9], Hod[7,8,9], Hpgd[7,9], Itm2a[7,8,9], Reck[7,9]
13	miR-221[10],miR-342[11]	Adh5[11], Atp6v1b2[10], Gas2[10], Ms4a4d[11], Polr2e[11], Rpo1-3[10]
14	miR-146[12],miR-181b[13],miR-181c[14]	Grik1[13,14], Klk6[12], Rex2[13,14], Svs6[12], V1ra2[13,14]
15	miR-155[15],miR-15a[16]	Kif22[16], Mgst1[15], Pla2g12a[16], Plekhc1[16], Wee1[15]
16	miR-150[17],miR-24[18]	Egr2[17], Mpo[17], Ngp[18], S100a8[18]

Table S 7: MicroRNA enrichment per cluster for `TCell` for `MixDTrees`

Cluster ID	MicroRNA	p-value
3	miR-222	0.0006906
5	miR-15a	0.0019456
	miR-26a	0.0369906
	miR-24	0.0369906
	miR-221	0.0051746
	miR-181a	0.0244306
7	miR-342	0.0200686
8	miR-26a	0.0013526
10	miR-150	0.0012176
	miR-142-3p	0.0000056
11	miR-16	0.0049776
	miR-146	0.0011936
	miR-181b	0.0049776

Table S 8: MicroRNAs enrichment per cluster for `BCell` for `MixDTrees`

Cluster ID	MicroRNA	*p*-value
3	miR-26a	0.0358116
	miR-181c	0.0025866
	miR-181b	0.0358116
5	miR-15b	0.0029956
	miR-15a	0.0029956
	miR-223	0.0029956
	miR-221	0.0323296
6	miR-191	0.0486736
	miR-155	0.0271276
19	miR-342	0.0402686
	miR-142-3p	0.0088346

Table S 9: MicroRNAs enrichment per cluster for `LTree` for `MixDTrees`

Cluster ID	MicroRNA	*p*-value
4	miR-26a	0.0003966
4	miR-342	0.0007606
6	miR-191	0.0069576
6	miR-150	0.0429006
7	miR-15b	0.0345666
11	miR-181c	0.0003136
11	miR-181a	0.0001926
11	miR-181b	0.0371576
13	miR-221	0.0223326
13	miR-342	0.0054556
14	miR-181c	0.0016926
14	miR-146	0.0047136
14	miR-181b	0.0003406
15	miR-15a	0.0067696
15	miR-155	0.0198496
16	miR-24	0.0032416
16	miR-150	0.0020206

Table S 10: MicroRNA enrichment per cluster for `TCell` for SOM

Cluster ID	MicroRNA	p-value
6	miR-15b	0.0080066
6	miR-15a	0.0130486
6	miR-26a	0.0439066
6	miR-221	0.0084286
15	miR-342	0.0000006
16	miR-16	0.0451586
16	miR-24	0.0000016
16	miR-181a	0.0136176
16	miR-181b	0.0025206
20	miR-222	0.0050756
20	miR-146	0.0081976

Table S 11: MicroRNAs enrichment per cluster for `BCell` for SOM

Cluster ID	MicroRNA	p-value
4	miR-26a	0.0017746
6	miR-223	0.0018216
6	miR-155	0.0474326
6	miR-221	0.0298266
7	miR-181c	0.0000746
7	miR-181a	0.0012986
7	miR-181b	0.0001736

Table S 12: Contingency Table comparing results from SOM (lines) against `MixDTrees` (columns) on `TCell`

	1	2	3	4	5	6	7	8	9	10	11	12	13	14	15	16	17	18	19	20
1	41	24	4	1	0	0	0	0	0	0	0	0	0	0	0	0	0	1	0	4
3	6	38	14	1	34	0	0	0	0	0	0	0	0	0	0	0	0	0	0	6
6	2	1	1	14	2	11	2	2	0	6	0	0	0	0	0	0	0	0	0	1
2	4	12	31	32	25	13	0	0	0	0	0	0	0	0	0	0	0	0	0	1
8	0	1	10	0	13	1	0	0	1	0	0	0	1	0	0	0	0	3	0	2
5	0	0	0	35	8	88	3	34	0	4	0	0	0	0	0	0	0	0	0	0
10	0	0	1	0	0	1	15	6	9	1	0	0	0	1	0	0	1	3	0	0
14	0	0	0	0	0	0	10	7	2	23	9	0	0	0	0	0	3	0	0	0
15	0	0	0	0	0	0	0	0	19	0	0	16	0	0	0	0	0	0	0	0
9	0	0	0	0	0	0	3	35	0	49	0	0	0	0	0	0	0	0	0	0
16	0	0	0	0	0	0	0	0	17	0	12	21	1	2	0	0	1	0	0	0
18	0	0	0	0	0	0	0	0	4	0	0	47	18	2	0	0	1	0	0	0
12	2	0	0	0	0	0	0	0	1	0	0	4	11	5	7	4	8	5	2	1
17	0	0	0	0	0	0	0	0	5	0	4	0	7	4	7	1	8	0	2	0
19	0	0	0	0	0	0	0	0	0	0	0	6	15	35	40	4	27	0	0	0
13	2	0	0	0	0	0	0	0	0	0	0	0	0	4	7	34	21	0	6	1
20	0	0	0	0	0	0	0	0	0	0	0	0	0	0	3	24	23	0	0	0
4	4	0	5	2	0	0	5	0	3	0	0	1	0	0	0	0	0	11	0	0
11	1	0	2	0	3	0	0	1	0	0	1	0	0	0	0	6	2	0	3	10
7	2	1	0	0	0	0	0	0	0	0	0	0	0	0	0	12	1	0	11	13

Table S 13: Contingency Table comparing results from SOM (lines) against `MixDTrees` (columns) on `BCell`

	1	2	3	4	5	6	7	8	9	10	11	12	13	14	15	16	17	18	19	20
1	52	0	0	0	0	4	5	4	0	0	0	3	0	0	0	0	0	0	0	0
4	0	20	12	10	0	4	0	0	1	14	0	0	0	0	0	0	0	0	0	0
7	0	6	64	5	25	2	0	0	22	0	0	0	0	0	0	0	0	0	0	0
2	14	3	8	4	2	40	0	0	0	0	0	1	0	0	0	0	0	0	0	0
15	0	0	1	0	0	1	1	0	0	2	0	0	0	0	0	0	0	0	0	0
6	0	7	43	5	10	42	2	0	3	0	0	0	0	0	0	0	0	0	0	0
5	4	1	0	0	0	1	4	1	0	2	1	0	0	0	0	0	0	0	0	0
3	5	3	0	1	0	5	0	7	0	5	0	0	0	0	0	0	0	0	0	0
8	0	7	3	0	0	0	0	0	9	13	0	0	1	0	0	0	0	0	0	0
9	0	1	1	10	0	0	0	7	1	17	0	0	3	0	0	0	0	0	0	0
16	0	0	0	0	0	0	4	1	0	0	4	2	0	2	1	1	18	0	0	0
20	1	0	0	0	0	1	1	3	0	0	7	14	0	0	0	0	0	0	0	0
10	0	0	0	3	0	0	0	0	8	6	0	0	18	0	0	0	0	0	0	1
12	0	0	0	0	0	0	0	7	0	0	0	0	2	6	18	2	6	1	0	1
13	0	0	0	0	0	0	0	1	0	0	1	0	0	0	20	13	1	0	0	8
14	0	0	0	0	0	0	0	2	0	0	2	0	0	0	3	28	3	8	4	6
17	0	0	0	0	0	0	0	0	0	0	1	0	0	1	3	25	24	0	0	3
19	0	0	0	0	0	0	2	0	0	1	5	12	0	0	0	4	0	35	18	0
18	0	0	0	0	0	0	0	0	0	0	14	9	0	0	0	24	8	3	18	0
11	0	0	0	0	0	0	0	3	0	1	0	0	14	1	19	0	1	0	0	8

Table S 14: Contingency Table comparing results from `MixDTrees-MAP` (lines) against `MixDTrees-MLE` (columns) on `TCell`

	1	2	3	4	5	6	7	8	9	10	11	12	13	14	15	16	17	18	19	20
1	0	0	42	0	0	0	0	0	0	0	0	1	0	0	0	4	0	28	0	0
2	0	18	0	0	0	41	0	1	0	0	0	0	0	0	0	58	0	0	0	0
3	0	28	28	0	0	0	0	0	29	0	0	0	0	0	0	14	0	0	0	0
4	0	1	0	0	0	0	0	0	0	5	0	17	0	5	0	3	0	0	0	0
5	1	1	0	0	0	74	5	83	0	0	0	0	0	8	0	0	0	0	0	0
6	0	0	0	0	0	3	0	0	0	0	0	9	0	9	0	21	0	0	0	0
7	0	0	0	0	0	0	0	0	1	0	0	0	37	0	0	0	0	2	0	0
8	6	12	5	0	0	0	0	1	1	0	0	4	1	1	0	1	0	0	0	0
9	0	0	0	0	0	0	46	39	0	0	0	0	0	1	0	0	0	0	1	0
10	0	1	0	0	0	0	9	2	0	0	0	0	0	13	0	0	0	0	13	0
11	6	0	0	0	0	0	0	0	11	0	0	2	8	0	1	0	0	1	0	0
12	0	0	0	2	0	0	0	0	0	11	0	0	0	0	14	0	0	0	0	23
13	0	0	0	0	0	0	0	0	0	0	18	0	32	0	5	0	0	1	0	19
14	6	0	0	0	2	0	8	0	0	0	0	0	0	2	0	0	3	0	33	0
15	0	0	0	0	17	0	0	0	0	1	0	0	0	0	0	0	0	0	17	0
16	0	0	0	0	40	0	0	0	0	0	0	0	0	0	0	0	10	0	4	0
17	3	0	0	1	0	0	0	0	0	1	0	0	0	0	7	0	25	0	1	0
18	0	0	0	40	24	0	0	0	0	0	0	0	0	0	4	0	4	0	0	0
19	0	0	0	122	0	0	0	0	0	0	0	0	0	0	0	0	5	0	0	0
20	0	0	0	1	0	0	0	0	0	0	48	0	0	0	0	0	1	0	0	0

Table S 15: Contingency Table comparing results from `MixDTrees-MAP` (lines) against `MixDTrees-MLE` (columns) on `BCell`

	1	2	3	4	5	6	7	8	9	10	11	12	13	14	15	16	17	18	19	20
1	0	11	0	0	0	0	0	0	0	0	0	0	19	0	0	1	24	0	21	0
2	0	3	0	0	0	0	0	0	0	0	0	1	0	0	0	44	0	0	0	0
3	0	117	0	0	0	0	0	0	0	0	0	0	0	0	0	15	0	0	0	0
4	0	5	0	0	0	0	0	1	0	0	0	7	0	0	0	25	0	0	0	0
5	0	19	0	0	0	0	0	0	0	0	0	0	0	0	0	0	19	0	0	0
6	0	55	0	0	0	0	0	0	0	0	0	0	0	0	0	2	43	0	0	0
7	0	0	0	4	0	0	3	0	0	0	2	0	10	0	0	0	0	0	0	0
8	0	0	0	0	0	0	1	2	0	9	1	0	6	3	5	0	0	3	6	0
9	0	13	0	0	0	0	0	6	3	0	6	5	0	0	0	10	1	0	0	0
10	0	0	0	0	0	0	0	30	0	0	0	27	0	0	0	3	0	0	1	0
11	0	0	0	5	3	15	0	0	0	0	0	0	0	4	0	0	0	0	0	8
12	0	0	0	6	0	5	2	0	0	0	0	0	5	0	0	0	0	0	0	23
13	0	0	0	0	0	0	0	1	27	10	0	0	0	0	0	0	0	0	0	0
14	0	0	0	0	0	0	0	0	0	1	0	0	0	1	4	0	0	4	0	0
15	29	0	0	0	0	0	0	0	0	0	0	0	0	0	0	0	0	35	0	0
16	45	0	0	0	52	0	0	0	0	0	0	0	0	0	0	0	0	0	0	0
17	8	0	0	0	31	18	0	0	0	0	0	0	0	4	0	0	0	0	0	0
18	2	0	1	0	12	17	4	0	0	0	0	0	0	11	0	0	0	0	0	0
1	0	0	0	0	13	27	0	0	0	0	0	0	0	0	0	0	0	0	0	0
20	8	0	2	0	1	0	0	0	0	3	0	0	0	3	1	0	0	9	0	0
